# Cytosolic calcium handling signature: integration with clinical predictors enhances prediction of post-operative atrial fibrillation

**DOI:** 10.1093/eurheartj/ehaf609

**Published:** 2025-09-09

**Authors:** Funsho E Fakuade, Judith Gronwald, Paulina Brandes, Yannic Döring, Tony Rubio, Fitzwilliam Seibertz, Maria Knierim, Issam H Abu-Taha, Aschraf El-Essawi, Ahmad Fawad Jebran, Bernhard C Danner, Hassina Baraki, Markus Kamler, Ingo Kutschka, Jordi Heijman, Dobromir Dobrev, Constanze Schmidt, Stefan M Kallenberger, Niels Voigt

**Affiliations:** Institute of Pharmacology and Toxicology, University Medical Centre Göttingen, Robert-Koch-Straße 40, 37075 Göttingen, Germany; DZHK (German Centre for Cardiovascular Research), Partner Site Lower Saxony, Robert-Koch-Straße 40, 37075 Göttingen, Germany; Cluster of Excellence ‘Multiscale Bioimaging: From Molecular Machines to Networks of Excitable Cells’ (MBExC), University Medical Centre Göttingen, Robert-Koch-Straße 40, 37075 Göttingen, Germany; Department of Cardiothoracic and Vascular Surgery, University Medical Centre Göttingen, Göttingen, Germany; Institute of Pharmacology and Toxicology, University Medical Centre Göttingen, Robert-Koch-Straße 40, 37075 Göttingen, Germany; DZHK (German Centre for Cardiovascular Research), Partner Site Lower Saxony, Robert-Koch-Straße 40, 37075 Göttingen, Germany; Department of Cardiology and Pneumology, University Medical Centre Göttingen, Göttingen, Germany; Institute of Pharmacology and Toxicology, University Medical Centre Göttingen, Robert-Koch-Straße 40, 37075 Göttingen, Germany; DZHK (German Centre for Cardiovascular Research), Partner Site Lower Saxony, Robert-Koch-Straße 40, 37075 Göttingen, Germany; Institute of Pharmacology and Toxicology, University Medical Centre Göttingen, Robert-Koch-Straße 40, 37075 Göttingen, Germany; DZHK (German Centre for Cardiovascular Research), Partner Site Lower Saxony, Robert-Koch-Straße 40, 37075 Göttingen, Germany; Institute of Pharmacology and Toxicology, University Medical Centre Göttingen, Robert-Koch-Straße 40, 37075 Göttingen, Germany; DZHK (German Centre for Cardiovascular Research), Partner Site Lower Saxony, Robert-Koch-Straße 40, 37075 Göttingen, Germany; Institute of Pharmacology and Toxicology, University Medical Centre Göttingen, Robert-Koch-Straße 40, 37075 Göttingen, Germany; DZHK (German Centre for Cardiovascular Research), Partner Site Lower Saxony, Robert-Koch-Straße 40, 37075 Göttingen, Germany; Cluster of Excellence ‘Multiscale Bioimaging: From Molecular Machines to Networks of Excitable Cells’ (MBExC), University Medical Centre Göttingen, Robert-Koch-Straße 40, 37075 Göttingen, Germany; DZHK (German Centre for Cardiovascular Research), Partner Site Lower Saxony, Robert-Koch-Straße 40, 37075 Göttingen, Germany; Department of Cardiothoracic and Vascular Surgery, University Medical Centre Göttingen, Göttingen, Germany; Institute of Pharmacology, West German Heart and Vascular Centre, University Duisburg-Essen, Essen, Germany; DZHK (German Centre for Cardiovascular Research), Partner Site Lower Saxony, Robert-Koch-Straße 40, 37075 Göttingen, Germany; Department of Cardiothoracic and Vascular Surgery, University Medical Centre Göttingen, Göttingen, Germany; DZHK (German Centre for Cardiovascular Research), Partner Site Lower Saxony, Robert-Koch-Straße 40, 37075 Göttingen, Germany; Department of Cardiothoracic and Vascular Surgery, University Medical Centre Göttingen, Göttingen, Germany; DZHK (German Centre for Cardiovascular Research), Partner Site Lower Saxony, Robert-Koch-Straße 40, 37075 Göttingen, Germany; Department of Cardiothoracic and Vascular Surgery, University Medical Centre Göttingen, Göttingen, Germany; DZHK (German Centre for Cardiovascular Research), Partner Site Lower Saxony, Robert-Koch-Straße 40, 37075 Göttingen, Germany; Department of Cardiothoracic and Vascular Surgery, University Medical Centre Göttingen, Göttingen, Germany; Department of Thoracic and Cardiovascular Surgery, University Hospital Essen, Essen, Germany; DZHK (German Centre for Cardiovascular Research), Partner Site Lower Saxony, Robert-Koch-Straße 40, 37075 Göttingen, Germany; Department of Cardiothoracic and Vascular Surgery, University Medical Centre Göttingen, Göttingen, Germany; Division of Medical Physics and Biophysics, Gottfried Schatz Research Centre, Medical University of Graz, Graz, Austria; Department of Cardiology, Maastricht University Medical Centre and Cardiovascular Research Institute Maastricht, Maastricht University, Maastricht, The Netherlands; Institute of Pharmacology, West German Heart and Vascular Centre, University Duisburg-Essen, Essen, Germany; Department of Medicine and Research Centre, Montreal Heart Institute and Universit́e de Montreal, Montreal, Quebec, Canada; Department of Integrative Physiology, Baylor College of Medicine, Houston, TX, USA; DZHK (German Centre for Cardiovascular Research), Partner Site Lower Saxony, Robert-Koch-Straße 40, 37075 Göttingen, Germany; Department of Cardiology and Pneumology, University Medical Centre Göttingen, Göttingen, Germany; Department of Cardiology, Heidelberg University Hospital, Heidelberg, Germany; DZHK (German Centre for Cardiovascular Research), Partner Site Heidelberg, Mannheim, Germany; Health Data Science Unit, University Hospital Heidelberg and Centre for Quantitative Analysis of Molecular and Cellular Biosystems (BioQuant), University of Heidelberg, Im Neuenheimer Feld 267, 69120 Heidelberg, Germany; Department of Medical Oncology, National Centre for Tumor Diseases, Heidelberg University Hospital, Im Neuenheimer Feld 460, 69120 Heidelberg, Germany; Institute of Pharmacology and Toxicology, University Medical Centre Göttingen, Robert-Koch-Straße 40, 37075 Göttingen, Germany; DZHK (German Centre for Cardiovascular Research), Partner Site Lower Saxony, Robert-Koch-Straße 40, 37075 Göttingen, Germany; Cluster of Excellence ‘Multiscale Bioimaging: From Molecular Machines to Networks of Excitable Cells’ (MBExC), University Medical Centre Göttingen, Robert-Koch-Straße 40, 37075 Göttingen, Germany

**Keywords:** Post-operative atrial fibrillation, Calcium handling, Cardiac surgery, Health data science, Prediction score, Personalised medicine

## Abstract

**Background and Aims:**

Atrial fibrillation (AF) is a prevalent complication after cardiac surgery, worsening patient outcomes. Considering the established role of Ca^2+^-handling abnormalities in AF pathogenesis, this study aimed to evaluate if integrating cytosolic Ca^2+^-handling measurements with clinical risk factors enhances the risk prediction of post-operative AF.

**Methods:**

Clinical data from 558 patients undergoing cardiac surgery without pre-existing AF from two centres were analysed. From 94 of these patients, atrial cardiomyocytes were isolated from collected right atrial appendages and Ca^2+^ handling (L-type Ca^2+^ current, intracellular Ca^2+^ concentration) was assessed using patch-clamp. The predictive performance of combining both clinical and single-cell Ca^2+^ handling parameters was tested using sequential feature selection and logistic regression models.

**Results:**

Single-cell Ca^2+^-handling parameters through cluster analysis correlated with post-operative AF development and several cardiac diseases. Integration of Ca^2+^-handling parameters into a new post-operative AF risk prediction model improved its predictive accuracy by increasing the areas under the receiver operating characteristic (ROC) curves from 0.69 to 0.71 in the training and 0.76 to 0.79 in the validation cohort. Systolic Ca^2+^ level, along with clinical parameters such as age, left atrial dilatation, valvular heart disease, impaired renal function, and serum magnesium, was identified as an independent risk factor for post-operative AF. Additionally, a predictive score for AF occurrence at discharge and during rehabilitation has been developed, with area under the curve (AUC) values of 0.84 and 0.71, respectively. Incorporating the occurrence of AF during the immediate post-operative period as an additional predictor significantly enhanced the prediction of AF at discharge, achieving an AUC value of 0.94.

**Conclusions:**

Integrating cellular Ca^2+^ handling signature with clinical predictors improves the prediction of post-operative AF, highlighting the potential of incorporating functional cellular data into clinical risk models.

## Structured Graphical Abstract

**Structured Graphical Abstract:**
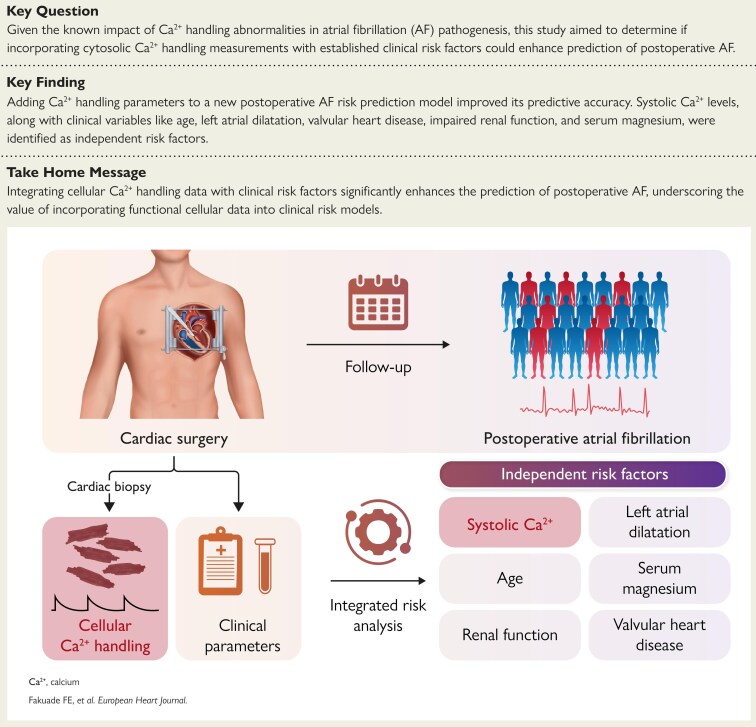
Clinical parameters were obtained from 558 patients undergoing cardiac surgery. Atrial cardiomyocyte cellular electrophysiology and Ca^2+^ was assessed in 94 of those patients. Patients were followed up and occurrence of AF was evaluated during the immediate post-operative (post-OP) period, at discharge and during rehabilitation. Systolic Ca^2+^ levels, along with age, left atrial dilatation, valvular heart disease, impaired renal function, and serum magnesium, were identified as independent risk factors.

**See the editorial comment for this article ‘Towards a personalized, mechanism-based risk prediction of post-operative atrial fibrillation’, by L. Giammarino and K.E. Odening, https://doi.org/10.1093/eurheartj/ehaf731.**

Translational perspective Atrial fibrillation is a common complication after cardiac surgery (post-OP AF) and is associated with poor outcomes. Identifying high-risk patients is essential for guiding prophylactic interventions. However, current prediction models have low sensitivity and modest accuracy. This study shows that pre-existing intracellular Ca^2+^-handling abnormalities in post-OP AF patients are an independent marker for post-OP AF risk and enhance clinical risk prediction scores. Thus incorporating detailed functional features of atrial cardiomyocytes may help to better capture pre-existing atrial cardiomyopathies, improving predictive-model accuracy. Advancements in automated methodologies, like automated patch-clamp techniques and Ca^2+^ imaging, show promise in facilitating the integration of cellular phenotypes into clinical studies and clinical risk prediction.

## Introduction

Atrial fibrillation (AF) notably remains the most common complicating adverse event occurring after cardiac surgery, with its incidence ranging between 10% and 60%.^1^ The varying incidence of this post-operative (post-OP) AF (post-OP AF) is attributable to the surgery type, employed monitoring technique and defined observation period.^2,3^ Despite advances in surgical strategies, post-OP AF prevalence has progressively increased over the decade, worsening patient clinical outcomes and health care system burden via increased mortality rates, prolonged hospital stays and elevated hospitalisation costs.^4–7^

Given the detrimental clinical and economic impact of post-OP AF, approaches to prevent this arrhythmia have been extensively investigated. Multiple studies have evaluated prophylactic strategies in preventing the development of post-OP AF.^8–10^ However, the success of these strategies is often overshadowed by the accompanying risks of side effects from utilised prophylactics and the potential of exposing a high percentage of patients with no indication of post-OP AF to these side effects.^3,10,11^ Hence, identifying high-risk patients within the patient pool is pertinent in limiting prophylactic and therapeutic interventions to these patient groups while excluding low-risk groups from the potential side effects and related costs.

To address this, several risk prediction models based on previously identified clinical risk factors, including age, obesity, valvular heart disease, lung disease and chronic kidney disease (CKD), to mention a few, have been developed to help predict post-OP AF risk accurately.^12–16^ However, the general predictive performance of most of these models remains modest, with area under the curve (AUC) values of the receiver operating characteristic (ROC) curves between 0.6 and 0.78, as shown in Supplementary data online, *Table S1*.^17^

Research has established that abnormalities in intracellular calcium (Ca^2+^) handling within cardiomyocytes are a key arrhythmogenic mechanism in various forms of AF, including paroxysmal and persistent AF as well as post-OP AF.^18–22^ During each heartbeat, Ca^2+^ enters cardiac myocytes through voltage-gated L-type Ca^2+^ channels (I_Ca,L_) and triggers a much larger Ca^2+^ release from the sarcoplasmic reticulum (SaR) by activating SaR Ca^2+^ release channels (cardiac ryanodine receptors, RyR2).^23^ This released Ca^2+^ binds to myofilaments to initiate cardiac contraction. During diastole, Ca^2+^ is pumped back into the SaR by ATP-dependent Ca^2+^-pump (SaR Ca^2+^ ATPase, SERCA2a) and extruded from the cell by Na^+^-Ca^2+^-exchanger, which brings three Na^+^ ions into the cell per extruded Ca^2+^ ion. Patients who develop post-OP AF have pre-existing alterations in this intracellular Ca^2+^ handling cycle, which may serve as a proarrhythmic substrate for surgery-related triggers that initiate AF.^24,25^ However, despite the well-observed existence of altered intracellular Ca^2+^ handling in AF patients, the added predictive value of incorporating these measurements into clinical risk prediction scores for AF has not been explored.

Therefore, considering the peculiar pre-existence of altered Ca^2+^ handling in patients who develop post-OP AF and the modest performance of currently available prediction models, we tested whether incorporating cellular Ca^2+^ handling parameters with clinical risk factors improves identifying patients at risk of developing AF not only during the immediate post-OP period, but also during discharge and rehabilitation periods following cardiac surgery.

## Methods

### Study population

Clinical data were collected from *n* = 558 patients without any history of AF before surgery. Patients underwent cardiac surgery at the Department of Thoracic and Cardiovascular Surgery of the University Medical Centre Göttingen (*n* = 535) between January 2017 and December 2020 as well as the Department of Thoracic and Cardiovascular Surgery of the University Hospital Essen (*n* = 23). The study was conducted in accordance with the Declaration of Helsinki and was approved by the ethics committee of the University Medical Centre Göttingen (no. 4/11/18) and University Hospital Essen (no. 12-5268-BO). Each patient gave written informed consent. An overview of the datasets used in this study is provided in Supplementary data online, *Figure S1*.

During the first three post-OP days, all patients were continuously monitored for their electrocardiogram (ECG). These ECGs were stored on the central monitoring systems of the intensive care unit (ICU), the intermediate care unit and the ward, after which they were analysed manually by an experienced clinician. Furthermore, a 12 lead-ECG was recorded on arrival to the ICU, on the first post-OP day, at discharge and when an arrhythmia was suspected. Patients were assigned to the post-OP AF group if any episode of AF lasting longer than 30 s was documented, while others were assigned to the control group (sinus rhythm, SR).

Additional information on clinical parameters is provided in the Supplementary Methods.

### Intracellular calcium measurements and cellular electrophysiology

Right atrial appendages were resected from *n* = 558 patients included in this study. A subset of 94 of these specimens was randomly selected for myocyte isolation, using a previously published standard protocol.^19,26^ To ensure the robustness of the dataset, the analysis of the 94 specimens encompassed cellular electrophysiological recordings, integrating both previously published data (147 cellular recordings from 79 patients)^24,25^ and unpublished data (108 cellular recordings from 54 patients). To assess the robustness of single-cell measurements, the variability of values between patients was compared with the uncertainty of measurements in single patients. In the case of patients where measurement was only possible in one cell (*n* = 18 of 71 patients), a linear error model was used to estimate the standard error. Evaluations showed that the variability between patients was, in most cases, larger than uncertainties of measurements in single patients (see Supplementary data online, *Figure S2*, see Supplementary  *Text S1* for details). Characteristics of the group of patients with available single-cell Ca^2+^ parameters are reported in Supplementary data online, *Table S2*. C-reactive protein (CRP) concentration was higher (by 18.7%), the tricuspid annular plane systolic excursion (TAPSE) was smaller (by 7.0%), and the post-OP potassium concentration was lower (by 2.1%) in the patient group with single-cell Ca^2+^ handling parameters compared with those without these parameters.

In brief, only rod-shaped myocytes with clear striations and defined margins were selected for measurements of the intracellular Ca^2+^ concentration ([Ca^2+^]_i_) and cellular electrophysiology. [Ca^2+^]_i_ of right atrial myocytes was measured using the fluorescent Ca^2+^ indicator Fluo-3 according to our previously published protocol.^19,24^ Whole-cell ruptured-patch-clamp technique (voltage-clamp) was used to record L-type Ca^2+^ current (I_Ca,L_) at 37°C and 0.5 Hz with simultaneous [Ca^2+^]_i_ measurement.^19,24^ Currents were normalised to membrane capacitance and expressed as current density (pA/pF).

### Statistical analysis

For comparisons of numeric or rank-ordered clinical parameters, the Wilcoxon rank sum test was applied. The flow chart visualising the AF status after surgery, at discharge or during rehabilitation treatment was created using SankeyMATIC. The presence of binary parameters was compared between groups using the Fisher exact test. Sequential forward selection was used to find optimal subsets of classification variables. Additional features were selected based on likelihood-ratio testing, assuming that the likelihood-ratio for a model including an additional variable compared with a model without the additional parameter follows a one-dimensional *χ*^2^ distribution. To assess classification performance, AUC values, sensitivities, specificities and classification accuracies were analysed. Statistical evaluations were conducted using MATLAB (The MathWorks, Natick MA, USA).

To identify the most predictive clinical parameters for AF or SR prediction after surgery, at discharge or during rehabilitation, clinical parameters were calibrated using multivariable logistic models. We assured that for all *n* = 79 clinical parameters tested as predictors, values were known in at least 60% of subjects. Before assessing the predictive value by sequential feature selection, a k-nearest neighbour imputation was performed for the dataset. An imputation was necessary for 6.6% of the clinical parameter values on average. For feature selection of binary parameters, strongly unbalanced parameters that were positive in <10 patients or negative in <10 patients were excluded. To evaluate the predictive performance of clinical datasets, 10-fold stratified cross-validation was used to determine ROC curves and their confidence intervals for pairwise classification between AF and SR.

Sequential feature selection and calibration of logistic regression models were further applied to assess the predictive performance of additionally combining *n* = 9 Ca^2+^ handling parameters [cellular capacitance (C_m_), diastolic [Ca^2+^]_i_, systolic [Ca^2+^]_i_, [Ca^2+^]_i_ transient (CaT) amplitude, CaT decay time, CaT time to peak, I_Ca,L_ peak, I_Ca,L_/C_m_, time integral of I_Ca,L_] with clinical parameters. Ca^2+^ handling parameters were obtained from measurements of 213 cardiomyocytes in 71 patients with available datasets of clinical parameters (between 1 and 8 cells per patient with a median of 3 cells per patient). By testing the predictive performance of additionally including Ca^2+^ handling parameters, a nested model structure was created. To analyse correlations between clinical parameters and single-cell measurements, means of Ca^2+^ parameters were calculated in subjects where multiple cardiomyocytes were measured. In the clinical parameter datasets of the patient group with available Ca^2+^ measurements, we again excluded binary parameters that were either positive or negative for <10 subjects. Applying this rule reduced the number of clinical parameters from 79 to 62. Accordingly, the feature selection started with a sum total of 71 clinical and Ca^2+^ handling parameters. For evaluating the predictive performance when using a combination of clinical and cellular Ca^2+^ handling parameters, 10-fold stratified cross-validation was performed. Additionally, different numbers *k* of *k*-fold cross-validation were tested. Differences of AUC values when including the most predictive Ca^2+^ handling parameter were assessed by performing *k*-fold cross-validation 100 times with random assignments to training and test datasets (see Supplementary data online, *Figure S3*).

For cluster analysis of clinical data and single-cell Ca^2+^ handling parameters measured in cardiomyocytes from atrial appendage samples, Spearman rank-order correlation coefficients were calculated for numeric or rank-ordered clinical parameters and Ca^2+^ handling parameters. Log2 fold changes were calculated to assess changes in single-cell Ca^2+^ handling parameters due to presence or absence of binary features. Groups with present or absent features were compared using one-way analysis of variance (ANOVA). Categorical variables between groups were compared with two-tailed Fisher exact test. The Benjamini-Hochberg method was used to correct for multiple testing. Hierarchical cluster trees were created using the complete-linkage clustering algorithm.

All data are incorporated into the article and its online supplementary material.

## Results

To assess if cytosolic Ca^2+^ handling data from atrial cardiomyocytes enhance post-OP AF prediction models, we performed an initial cluster analysis. This unbiased approach aims to identify patterns and correlations of cytosolic Ca^2+^ handling with cardiac diseases and conditions linked to post-OP AF. This is followed by a detailed examination of the predictive performance of combining clinical and cellular parameters, utilising logistic regression and sequential feature selection to refine our model's accuracy. We validated our enhanced model across multiple patient cohorts to ensure its robustness and generalisability in predicting post-OP AF. Finally, to illustrate the potential added value of integrating cellular Ca^2+^ handling data, we present an analysis using purely clinical data from patients who underwent cardiac surgery during the study period.

### Clusters of clinical and calcium handling parameters indicate associations with cardiac diseases

**Figure 1:**
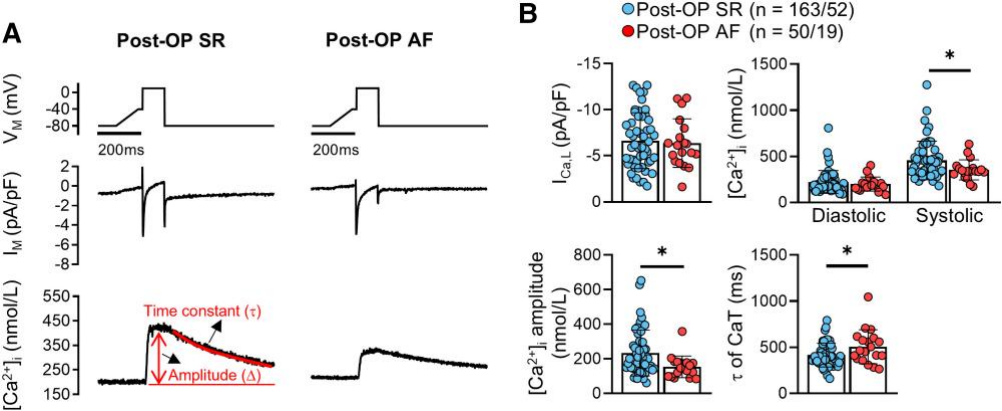
Calcium (Ca^2+^) handling parameters in atrial cardiac myocytes from patients developing post-operative AF. (*A*) Voltage-clamp protocol (0.5 Hz, top), alongside representative recordings of simultaneous L-type Ca^2+^ current (I_Ca,L_, middle) and resulting triggered Ca^2+^ transients (CaT, bottom) in sinus rhythm (post-OP SR) and post-operative AF (post-OP AF) atrial myocytes. (*B*) Peak I_Ca,L_ (top-left) along with diastolic and systolic [Ca^2+^]_i_ (top-right) of corresponding triggered CaT. Calculated amplitudes (Δ[Ca^2+^]_i_, bottom left) and time constants of decay (τ, bottom right) of I_Ca,L_-triggered CaT. Data are presented as mean ± standard deviation. *P < .05 vs post-OP SR. *n* = number of myocytes/patients. Each dot represents the mean value of cellular recordings obtained from an individual patient. Comparisons using one-way ANOVA rank sum test (see Supplementary data online, *Table S3* for details).

As previously reported, I_Ca,L_ was comparable in both post-OP AF and post-OP SR patients. The systolic [Ca^2+^]_i_ was significantly lower in post-OP AF despite comparable diastolic values, resulting in smaller CaT amplitude (*Figure 1*). Determination of the time constant *τ* of CaT decay by the exponential fitting of the decay phase of the CaT revealed a significantly larger time constant in post-OP AF compared with post-OP SR, indicating delayed removal of cytosolic Ca^2+^ during systole in post-OP AF. Comparisons of all 9 measured single-cell parameters between post-OP AF and SR are documented in Supplementary data online, *Table S3*.

**Figure 2:**
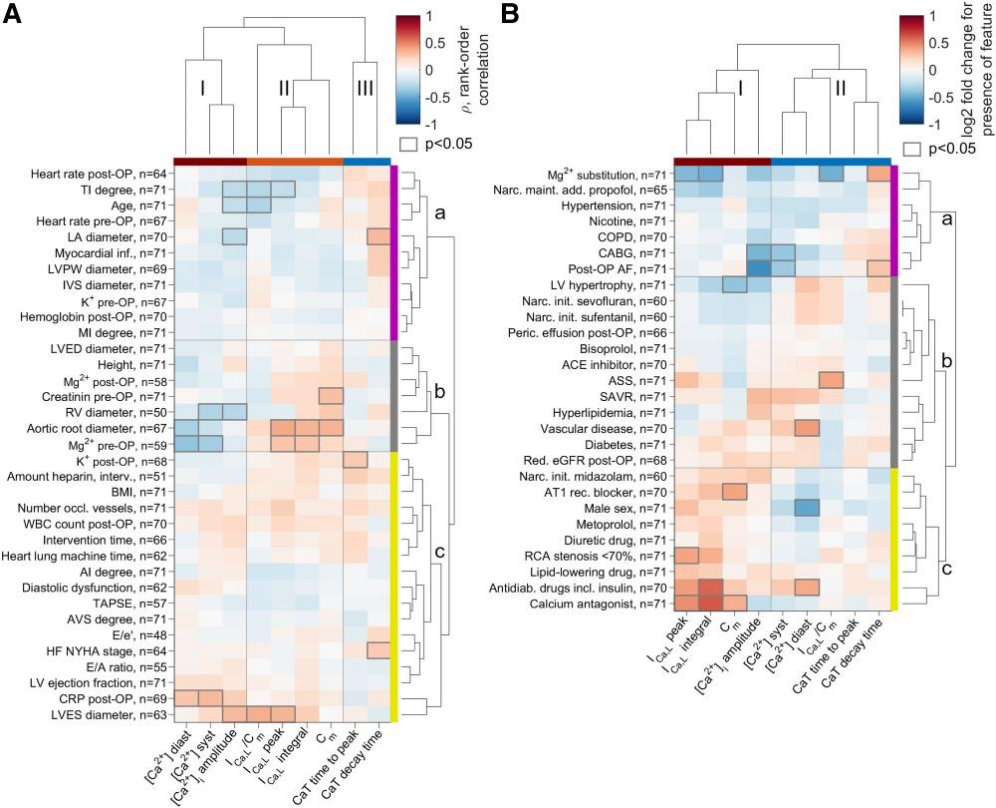
Clusters of clinical parameters and calcium (Ca^2+^)-handling parameters indicate physiological implications in different types of cardiac diseases. (*A*) Clusters of correlation coefficients between continuous or rank-ordered clinical parameters and Ca^2+^ measurements. Clinical parameters were grouped in three clusters a, b, and c, according to correlations with Ca^2+^ handling parameters as indicated by coloured bars (purple, grey, and yellow). Ca^2+^-handling parameters were grouped to three clusters I, II and III (maroon, orange, and blue). (*B*) Clusters of binary clinical parameters a, b, and c (purple, grey, and yellow) with Ca^2+^ handling measurements I and II (maroon, blue) based on log2 fold changes in Ca^2+^ parameters for presence compared with absence of the binary parameters. Numbers of patients, in which clinical parameters were available, are indicated. ACE, angiotensin converting enzyme; AI, aortic valve insufficiency; AVS, aortic valve stenosis; ASS, acetylsalicylic acid treatment; AT1 rec., angiotensin receptor 1; BMI, body-mass index; CABG, coronary artery bypass grafting; COPD, chronic obstructive pulmonary disease; CRP, C-reactive protein; E/e′, ratio between E-wave and e′ velocity as measured by echocardiography; E/A, ratio between E-wave and A-wave as measured by echocardiography; HF, heart failure; IVS, interventricular septum; LVPW, left ventricular posterior wall; LA, left atrium; LV, RV, left/right ventricle; LVED, left ventricular end-diastolic; LVES, left ventricular end-systolic; MI, mitral valve insufficiency; narc. maint. add. propofol, additional administration of propofol for maintenance of narcosis; narc. init., narcosis initiation; occl., occluded; peric., pericardial; RCA, right coronary artery; red., reduced; SAVR, surgical aortic valve replacement; TAPSE, tricuspid annular plane systolic excursion; TI, tricuspid valve insufficiency; WBC count, white blood cell count; [Ca^2+^]_diast._, diastolic [Ca^2+^]_i_; [Ca^2+^]_syst._, systolic [Ca^2+^]_i_. Grey boxes indicate *P* < .05, in (*A*) from testing for Spearman rank order correlation, in (*B*) from one-way ANOVA

To systematically analyse groups of medical and single-cell parameters associated with disease phenotypes and to study associations between clinical parameters and single-cell Ca^2+^ parameters, we performed cluster analyses (*Figure 2*). The cluster analysis visualises which clinical parameters are associated with Ca^2+^-handling parameters in a similar manner. Clusters are separated based on a linkage function that quantifies the dissimilarity of the vectors of correlation coefficients. It provides an overview of the whole set of clinical and Ca^2+^-handling parameters.

In *Figure 2A*, correlation coefficients are visualised on a colour scale, clusters are highlighted by colour bars (purple, grey, yellow for clinical parameters, as well as maroon, orange, and blue for Ca^2+^ parameters). The cluster analysis of associations between continuous or rank-ordered clinical features with single-cell Ca^2+^-handling parameters was conducted based on Spearman rank-order correlation coefficients with Ca^2+^-handling parameters. Grey squares indicate correlations with *P* < .05. Following correction for multiple testing (Benjamini-Hochberg), none of the correlations remain significant. Clinical parameter clusters could be associated with pathophysiological characteristics of patient groups.

The first cluster (cluster a/purple in *Figure 2A*) contains the parameters mitral valve insufficiency degree and tricuspid valve insufficiency (TI) degree as well as parameters associated with dilatation or enlargement of cardiac compartments [left atrium (LA), left ventricular posterior wall (LVPW) and interventricular septum (IVS) diameters]. As part of this cluster, negative correlations can be observed between the clinical parameters LA diameter, age and TI degree, and the CaT amplitude, the I_Ca,L_ current normalised by the C_m_, and the peak I_Ca,L_ current. Further, the LA diameter is positively correlated with the CaT decay time, i.e. with a slower CaT decay.

The second clinical parameter cluster (cluster b/grey in *Figure 2A*) contains the parameters aortic root diameter, left ventricular end-diastolic (LVED) diameter and right ventricular (RV) diameter that can be associated with aortic valve stenosis (AVS) and cardiac hypertrophy. As part of this cluster, the clinical parameters RV diameter, the aortic root diameter and the pre-operative Mg^2+^ concentration are negatively correlated with the diastolic and systolic [Ca^2+^]_i_ as well as the CaT amplitude. Further, the aortic root diameter and the preoperative Mg^2+^ concentration are positively correlated with the I_Ca,L_ current peak, the I_Ca,L_ current integral and the C_m_, an index for cardiomyocyte size.

The third cluster (cluster c/yellow in *Figure 2A*) comprises clinical parameters associated with heart failure (HF), arteriosclerosis, inflammation, coronary heart disease or cardiac decompensation, such as number of occluded coronary vessels, white blood cell count, diastolic dysfunction, New York Heart Association (NYHA) HF stage, left ventricular (LV) ejection fraction or CRP. Parameters of this cluster are linked to increased intervention risk. Within this cluster, positive correlations can be observed between CRP and systolic [Ca^2+^]_i_ as well as diastolic [Ca^2+^]_i_, between NYHA HF stage and the CaT decay time, and further between the left ventricular end-systolic (LVES) diameter and the Ca^2+^ concentration amplitude, the I_Ca,L_ peak current and the peak I_Ca,L_ current normalised by the C_m_.

*Figure 2B* visualises a cluster analysis of binary clinical parameters and cellular Ca^2+^ measurements. The cluster analysis was performed based on log2 fold changes in single-cell Ca^2+^ parameters due to presence or absence of binary clinical parameters. Groups with present or absent clinical parameters were compared using one-way ANOVA. The first cluster (cluster a/purple in *Figure 2B*) contains the parameters post-OP Mg^2+^ substitution and post-OP AF besides chronic obstructive pulmonary disease (COPD), smoking and hypertension that can be associated with cardiovascular risk. As part of this cluster, Mg^2+^ substitution is associated with decreased peak and integral of the I_Ca,L_ current, decreased peak I_Ca,L_ current normalized by the C_m_ and a slower CaT decay. Coronary artery bypass surgery and post-OP AF are associated with decreased systolic [Ca^2+^]_i_ and a decreased CaT amplitude.

The second cluster (cluster b/grey in *Figure 2B*) comprises parameters that can be linked to metabolic and vascular disorders. As part of this cluster, in patients with acetylsalicylic acid treatment, an increased peak I_Ca,L_ current normalised by C_m_ and in patients with vascular diseases, an increased diastolic [Ca^2+^]_i_, were observed.

Finally, the third cluster (cluster c/yellow in *Figure 2B*) contains parameters associated with treatment of hypertension and coronary artery disease (CAD) [angiotensin receptor 1 (AT1) blocker, metoprolol, diuretic drugs, lipid-lowering drugs, right coronary artery (RCA) stenosis ≥ 70%, Ca^2+^-channel antagonist]. As part of the third cluster, the presence of an RCA-stenosis > 70% and the intake of Ca^2+^ channel antagonists are associated with an increased peak I_Ca,L_ current. Intake of Ca^2+^ channel antagonists is further associated with increased integral of the I_Ca,L_ current and increased C_m_. The intake of antidiabetic drugs, including insulin, is related to an increased I_Ca,L_ current integral and increased diastolic [Ca^2+^]_i_. Further, male sex was linked to a decreased diastolic [Ca^2+^]_i_.

### Combining a calcium handling parameter with clinical parameters improves the predictability of post-operative AF

**Figure 3:**
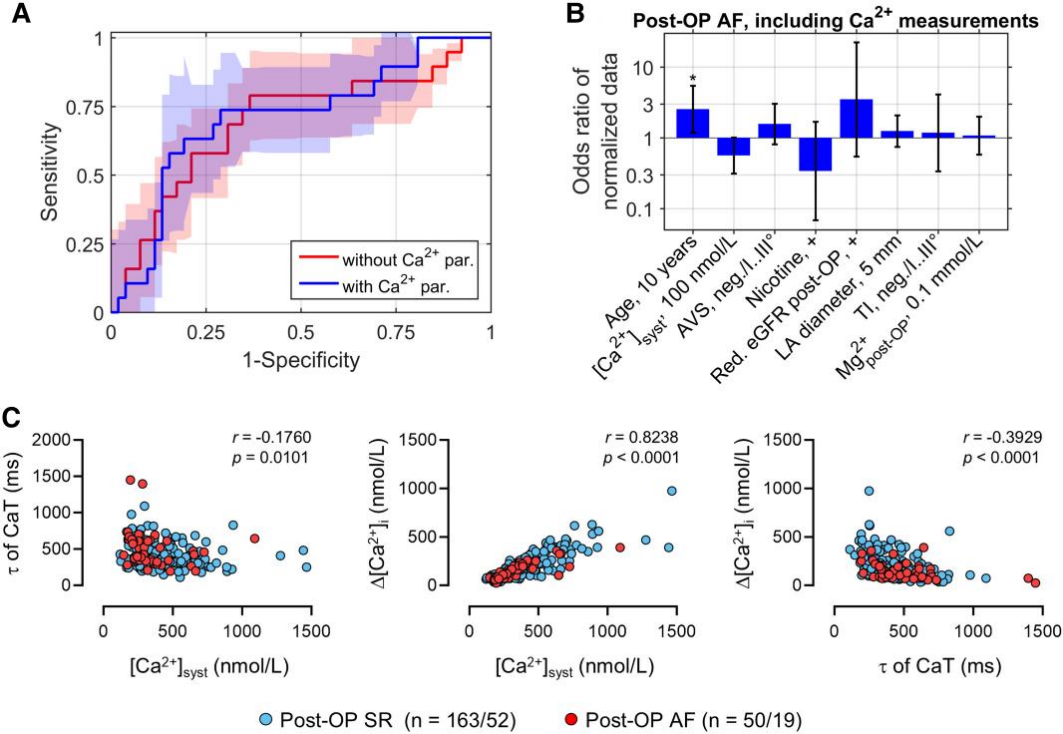
Risk prediction for development of atrial fibrillation (AF) following cardiac surgery using clinical and cellular calcium (Ca^2+^)-handling parameters. (*A*) ROC curves for post-OP AF prediction without [AUC = 0.69; 95% CI: (0.58, 0.81)] or with additional Ca^2+^ handling parameter [AUC = 0.71; 95% CI: (0.61, 0.81); areas: 95% confidence intervals from 10-fold cross-validation]. Due to correlations between Ca^2+^ parameters, including a second Ca^2+^ parameter did not further improve AF prediction. (*B*) Bars indicate odds ratios of clinical parameters and systolic [Ca^2+^]_i_ ([Ca^2+^]_syst._) i.e., ratios between the chance of developing AF to the chance of not developing post-OP AF (error bars: 95% confidence intervals; *P < .05, see Supplementary data online, *Table S4* for details). Parameters were sorted from small (left) to larger *P*-values (right). Odds ratios reflect the effects of binary variable changes, indicated by a ‘+’ or continuous variable changes by indicated unit intervals or numbers *n*. (*C*) Correlation between time constants of decay (τ) and amplitudes (Δ[Ca^2+^]) of L-type Ca^2+^ current (I_Ca,L_)-triggered CaT in SR and post-OP AF atrial myocytes. *r* = Pearson’s correlation coefficient; *n* = number of myocytes/patients. AVS, aortic valve stenosis; LA, left atrium; TI, tricuspid valve insufficiency

The observed differences between post-OP AF and post-OP SR at the single-cell level led us to test the predictive value of measurements in atrial cardiomyocytes in conjunction with clinical parameters. To this end, means of Ca^2+^ measurements from 213 cardiomyocytes of 71 patients with available medical parameter datasets were calculated. Several clinical parameters are causally linked to pathophysiological changes in the heart that can result in the development of post-OP AF.^1^ Sequential feature selection from the combination of clinical parameters and a set of 9 Ca^2+^-handling parameters followed by 10-fold cross-validation showed a slight improvement of the predictive accuracy, as demonstrated by the increase of the AUC value of ROC curves for post-OP AF prediction from 0.69 [95% CI: (0.58, 0.81)] to 0.71 [95% CI: (0.61, 0.81)] (*Figure 3A*, Supplementary data online, *Table S4*). *Figure 3B* indicates odds ratios of parameters predictive for post-OP AF, obtained from sequential feature selection. Parameters were ordered by increasing *P*-values. The most predictive parameters for predicting post-OP AF were age and low systolic [Ca^2+^]_i_, the only Ca^2+^ handling parameter included as a predictor for post-OP AF. The increase in predictive accuracy, assessed by AUC values of ROC curves, from including this parameter was robust to the number of cross-validations (see Supplementary data online, *Figure S3*). The CaT decay constant *τ* and the CaT amplitude, which differed between SR and AF groups (*Figure 1B*), were not included as predictors due to correlations with the systolic [Ca^2+^]_i_ (*Figure 3C*). Moreover, the degree of AVS and reduced estimated glomerular filtration rate (eGFR) were predictive for post-OP AF as well as LA diameter, degree of TI and post-OP serum concentration of Mg^2+^. Smoking was related to a decreased risk for post-OP AF, probably reflecting separate groups of cardiac pathologies associated with different risks for post-OP AF. Taken together, these findings established that single-cell Ca^2+^ measurements in human atrial cardiomyocytes possess a predictive value for the occurrence of post-OP AF.

The score was tested in an independent dataset of single-cell systolic [Ca^2+^]_i_ of isolated human atrial cardiomyocytes together with the selected clinical parameters (age, LA diameter, post-OP Mg^2+^ serum concentration, reduced eGFR post-OP, smoking status, AVS degree, TI degree) in *n* = 23 patients.^25^ We tested the score under possible combinations of assumptions to adapt datasets regarding the smoker status (current, past, present), the AVS stage and status after surgical AVS treatment, which was the case in 12 of 23 patients in the study by Heijman *et al.*,^25^ and applied batch correction factors due to significant differences in age, post-OP Mg^2+^ concentration, the degree of AVS and systolic [Ca^2+^]_i_ of isolated human atrial cardiomyocytes. In all cases, including the single-cell CaT parameters improved the prediction of post-OP AF. When applying batch correction factors adjusting for differences in parameter means, assuming (i) that the status ‘ex-smoker’ was equivalent to ‘no smoker’ and (ii) that ‘after surgical AVS treatment’ was equivalent to ‘severe AVS’, including the additional single-cell CaT parameters resulted in an AUC increase from 0.76 to 0.79 (see Supplementary  *Text S2* for details).

### Predictability of post-operative AF using clinical data only

To compare our risk score, which incorporates experimental Ca^2+^-handling parameters along with clinical data, with scores derived solely from clinical data, we analysed the clinical data of all patients who underwent cardiac surgery during the study period.

**Figure 4:**
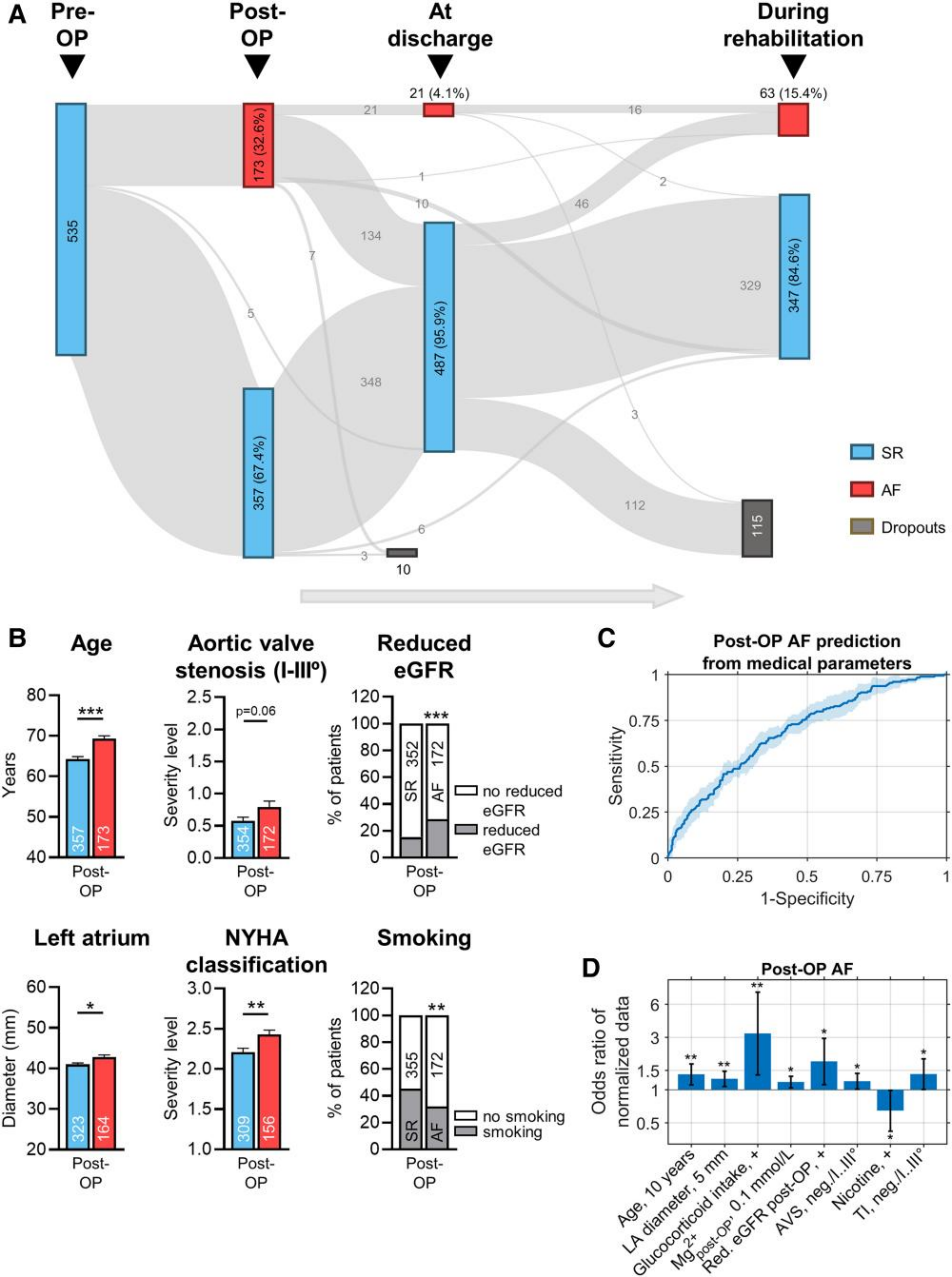
Risk prediction for development of atrial fibrillation following cardiac surgery (post-OP AF). (*A*) AF status of patients during the post-operative period, discharge and rehabilitation periods after cardiac surgery. (*B*) Selected clinical characteristics of patients who developed post-OP AF opposed to patients with sinus rhythm. Data are presented as mean ± standard deviation or percentages of patients. *P < .05, **P < .01, ***P < .001 vs SR. Comparison using Wilcoxon rank sum test (see Supplementary data online, *Table S5* for details). eGFR, estimated glomerular filtration rate; NYHA, New York Heart Association. (*C*, *D*) Logistic regression model calibrated using medical history and clinical parameters. Predictive parameters were selected based on sequential feature selection. (*C*) ROC curve for predicting post-OP AF [AUC = 0.69; 95% CI: (0.66, 0.72); areas: 95% confidence intervals from 10-fold cross-validation]. (*D*) Bars indicate odds ratios, i.e., ratios between the chance of developing AF to the chance of not developing post-OP AF (error bars: 95% confidence intervals; *P < .05, **P < .01, ***P < .001, see Supplementary data online, *Table S9* for details). Parameters were sorted from small (left) to larger *P*-values (right). Odds ratios reflect the effects of binary variable changes, indicated by a ‘+’ or continuous variable changes by indicated unit intervals or numbers *n*. AVS, aortic valve stenosis; LA, left atrium; TI, tricuspid valve insufficiency

A total of 535 patients who underwent cardiac surgery were included in the analysed period of the study. Enrolled patients had a mean age of 65.8 years (20 to 85 years) and were predominantly male (84% of the total patient population). Collectively, in 442 patients (82.6%), an aortocoronary bypass grafting was performed, and 147 patients (27.5%) underwent an aortic valve replacement. There was an intersection of 11.2% of patients undergoing both interventions. A mitral valve reconstruction was performed in *n* = 7 patients (1.3%) and a tricuspid valve reconstruction in 2 patients (0.4%). 32.6% of the patients developed AF in the in-hospital post-OP period, i.e. within 3 to 6 days after cardiac surgery (post-OP AF, *Figure 4A*). Until discharge, 86% of patients who had post-OP AF returned to SR (134 of 155 patients with known status of SR or AF at discharge), while AF remained in 14% of the patients. In 30% of patients with AF during rehabilitation, AF was not diagnosed in the in-hospital post-OP period. Moreover, in 73% of the patients with AF during rehabilitation, AF was not observed during discharge (*Figure 4A*, and Supplementary  *Text S3*).

Patients who experienced post-OP AF had significantly different clinical parameters compared with patients with post-OP SR, as they were significantly older, more frequently had lower glomerular filtration rates (eGFR < 60 mL/min/1.73 m^2^) and higher HF level (NYHA classification) compared with patients with post-OP SR (*Figure 4B*). In addition, LA diameter was also increased in post-OP AF patients. Among patients with history of smoking, post-OP AF was less frequent. Baseline characteristics and all comparisons between post-OP AF and post-OP SR are listed in Supplementary data online, *Tables S5*–*S7*. Further comparing clinical characteristics between patients with post-OP AF, AF at discharge or during rehabilitation indicated an over-representation of patients with AVS in the group of patients with AF at discharge or at rehabilitation (see Supplementary data online, *Table S8*, see Supplementary  *Text S3* for details).

Subsequently, we analysed the predictive value of 79 different clinical parameters (medical history, laboratory values, drug intake, modalities of cardiac surgery and anaesthesia, echocardiographic parameters) for the development of post-OP AF. Logistic regression models predicting either SR or AF were calibrated using the set of clinical parameters. Parameters were selected based on sequential feature selection. According to the ROC curve, testing with 80% sensitivity would result in a specificity of about 44% (*Figure 4C*). The accuracy of predicting AF from clinical data increased from the time immediately after surgery [ROC curve with AUC 0.69; 95% CI: (0.66, 0.72)] to the time at discharge [AUC 0.84; 95% CI: (0.73, 0.95)], and again decreased to the time during rehabilitation treatment [AUC 0.71; 95% CI: (0.65, 0.76)]. Finally, we observed that including post-OP AF as additional predictor for AF at discharge, or including post-OP AF and AF at discharge for predicting AF during rehabilitation results in improved performance. Including post-OP AF for predicting AF at discharge resulted in an increase of the AUC from 0.84 to 0.94 [95% CI: (0.92, 0.97)]. Further, including post-OP AF and AF at discharge as predictors for AF during rehabilitation resulted in an increase of the AUC from 0.71 to 0.76 [95% CI: (0.70, 0.83); see Supplementary  *Text S4* and Supplementary data online, *Figure S3* for details].

*Figure 4D* presents odds ratios of parameters predictive for post-OP AF, obtained from sequential feature selection and arranged in order of increasing *P*-values. Parameters were similar to post-OP AF prediction relying on combined Ca^2+^ handling and clinical parameters (*Figure 3B*). In the clinical data only prediction model, the parameter ‘glucocorticoid intake’ replaced the parameter ‘[Ca^2+^]_syst._’ as a predictor and was related to ∼3.2-fold higher odds for post-OP AF (*Figure 4D*). The most predictive parameters for predicting post-OP AF were age and LA diameter, followed by glucocorticoid intake, post-OP serum concentration of Mg^2+^ and reduced eGFR, with reduced eGFR having 1.8-fold higher odds. Moreover, degrees of AVS and TI were predictive for post-OP AF, whereas smoking was related to decreased risk for post-OP AF. Values of all model parameters, odds ratios and *P*-values are provided in Supplementary data online, *Tables S9*–*S13*.

## Discussion

In the present study, we thoroughly analysed clinical and experimental parameters in SR patients undergoing cardiac surgery, both with and without subsequent development of AF. For the first time, our analysis expanded to cellular electrophysiology and Ca^2+^ handling data from human cardiomyocytes isolated from right atrial appendages obtained during the surgical procedure. Through this approach, we identified the concentration of systolic Ca^2+^ in atrial cardiomyocytes as an independent risk marker for development of post-OP AF. Consistent with previous studies (see Supplementary data online, *Table S1*), we identified age, obesity, atrial dilatation, valvular heart disease and impaired renal function as independent parameters increasing the risk for the development of post-OP AF. Further patient monitoring revealed that the emergence of AF in the immediate post-OP period until hospital discharge strongly predicts AF development during rehabilitation.

### Abnormal cytosolic Ca^2+^ handling points to pre-existing atrial cardiomyopathies

**Figure 5:**
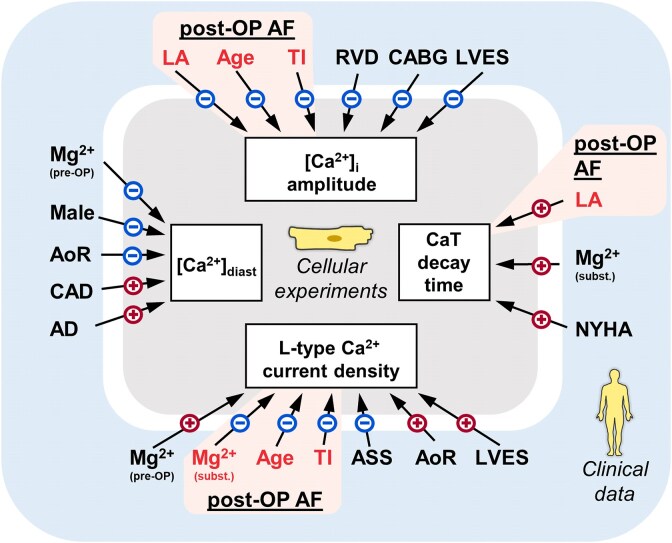
Schematic overview of clinical parameters and their effect on cytosolic calcium (Ca^2+^) handling. Mechanisms and clinical factors potentially involved in remodelling of cytosolic Ca^2+^ handling predisposing to post-OP AF are highlighted in red shading. AD, antidiabetic drug treatment; AoR, aortic root diameter; ASS, acetylsalicylic acid treatment; CABG, coronary artery bypass grafting; CAD, coronary artery disease; CaT, Ca^2+^ transient; LA, left atrial diameter; LVES, left ventricular end-systolic diameter; NYHA, New York Heart Association classification of heart failure; RVD, right ventricular diameter; TI, tricuspid valve insufficiency; [Ca^2+^]_diast._, diastolic [Ca^2+^]_i_; [Ca^2+^]_syst._, systolic [Ca^2+^]_i_

We provide the first comprehensive cluster analysis revealing associations between clinical parameters and atrial cardiomyocyte Ca^2+^-handling parameters (*Figure 5*). Interestingly, our unbiased analysis revealed remarkable similarities to previous studies in human and animal models, which were explicitly designed to investigate the interaction between the respective clinical parameters and atrial Ca^2+^ handling. The overlap with previously identified factors in individual models underscores their relevance in the clinical setting, and we will therefore discuss this association in more detail in the following paragraphs.

Age is the strongest predictor of AF, including post-OP AF.^1,15^ Consistent with our findings, previous studies have reported reduced I_Ca,L_, diminished Ca^2+^ reuptake into the SaR by SERCA2a, and lower systolic Ca^2+^ release in older patients.^27^ Given our prior identification of reduced SERCA2a activity as a key factor in post-OP AF development,^24^ we postulate that age-related decline in SERCA2a activity may contribute to its development.

In addition, chronic atrial pressure or volume overload due to valvular heart disease appear to be associated with reduced I_Ca,L_ and SERCA activity, which were previously associated with increased susceptibility to atrial arrhythmias.^19,24,28–30^ Accordingly, our data suggest that I_Ca,L_ and systolic Ca^2+^ release are reduced in patients with valvular heart disease (particularly in TI), increased right ventricular diameter and in case of an increased LA diameter.

Mg^2+^ supplementation is frequently discussed as a potentially beneficial intervention to prevent or treat AF, though the supporting data remain inconclusive.^31^ The interest in Mg^2+^ as a potential antiarrhythmic agent is largely based on associations between low serum Mg^2+^ levels and the occurrence of AF.^32,33^ The antiarrhythmic potential of Mg^2+^ may be due to inhibition of potassium, sodium^34^ and Ca^2+^ channels.^35^ The latter is in accordance with our observation that post-OP Mg^2+^ supplementation, pointing to low Mg^2+^ levels at the time of surgery, is associated with increased I_Ca,L_. Antiarrhythmic effects of Mg^2+^ have also been proposed to involve RyR2 receptor stabilization and reduced diastolic SaR Ca^2+^ leak.^36^ Indeed, our data reveal a negative correlation between pre-operative serum Mg^2+^ levels and diastolic Ca^2+^ levels, that are largely determined by diastolic SaR Ca^2+^ leak. However, Mg^2+^ supplementation does not necessarily achieve successful cardioversion or reliable AF prevention.^33,37^ This suggests a causal disconnect between Mg^2+^ and antiarrhythmic effects, with additional confounding factors likely contributing to the observed link between hypomagnesemia and arrhythmias.

In our cohort, increased diastolic Ca^2+^ levels were also associated with female sex and diabetes, the latter representing an established risk factor for AF. Again, it is likely that higher diastolic Ca^2+^ levels are caused by diastolic RyR2 leak as seen in models of diabetic cardiomyopathy^38^ and recent findings of increased Ca^2+^ sparks in female atrial myocytes.^39^ However, future studies are necessary to determine the role of increased SaR Ca^2+^ leak in atrial arrhythmogenesis in female patients and diabetes.^40^

Taken together, this analysis provides insights into potential mechanisms underlying atrial cardiomyopathy in specific patient cohorts and may provide a step toward developing personalised prevention and therapeutic strategies.^41^ Although these results need to be interpreted with caution because of potential uncontrolled confounding factors, it is important to note that many parameters identified to influence cytosolic Ca^2+^ homeostasis, such as age, LA diameter and valvular heart disease, were also predictive markers for post-OP AF development.

### Clinical risk factors of post-operative AF

Clinical risk factors identified in our present analysis, along with previously published risk scores (see Supplementary data online, *Table S1*), have been associated with the occurrence of post-OP AF. These risk factors are thought to drive specific atrial remodelling that promotes the initiation and maintenance of AF.^20^ Mechanisms involving remodelling of cytosolic Ca^2+^ handling are discussed above and highlighted in red shading in *Figure 5*. Additional molecular mechanisms underlying the increased AF susceptibility in presence of these factors have been studied extensively during the last decades and are summarised below.

Advanced age is one of the risk factors most strongly associated with AF development. Advanced age increases conduction time and atrial electrical heterogeneity, promoting AF occurrence.^15,42,43^ Although advanced age is a non-modifiable risk factor, DNA-based markers of accelerated biological ageing have been shown to be independently associated with AF.^44^ In addition, recent studies demonstrate that senolytic therapy reduces AF susceptibility in a rat model of myocardial infarction.^45^ Taken together, these data suggest that influencing biological ageing through targeting lifestyle may be a promising approach for AF prevention.

Valvular heart disease has been associated with a 1.8- and 3.4-fold increased risk for AF in men and women, respectively.^15^ Valvular heart disease leads to chronic pressure or volume overload, depending on the type of valvular lesion. The resulting mechanical stretch initiates pathways that cause fibrosis and alter myocyte coupling and function, resulting in structural and electrical remodelling.^46,47^ We also identified increased LA diameter as an independent predictor of post-OP AF. In addition to valvular heart diseases, LA dilatation may be caused by other pathological entities increasing atrial load, such as HF and hypertension, which have been previously reported as risk factors for post-OP AF development.^48^

Preoperative glucocorticoid administration has been suggested to prevent post-OP AF in high-risk patients.^49,50^ The preventive effect of glucocorticoids has been attributed to the inhibition of cytokine release e.g. Tumor necrosis factor alpha (TNF-α) and interleukin 6 (IL-6), and subsequent reduction in the activation of the complement system.^49,50^ However, previous studies have shown that prednisolone treatment increased AF inducibility in a canine model of sterile pericarditis.^51^ The authors suggest this may be due to the increased expression of small-conductance Ca^2+^-activated K^+^ (SK) channels,^52^ which are known to be modulated by glucocorticoids.^53^ Our data align with an arrhythmogenic effect of glucocorticoid treatment. However, glucocorticoid use may also represent a marker for pre-existing inflammatory conditions and pulmonary diseases that have been shown previously to promote the occurrence of post-OP AF.^1,13,54^

Finally, in previous studies, AF is more prevalent in patients with CKD, even after accounting for other associated risk factors such as HF, CAD and hypertension. AF risk increases with severity of renal dysfunction, pointing to a potential causative relation.^16^ Therefore, several mechanisms have been proposed to underlie AF development in patients with CKD, including increased inflammation^55^ and activation of the renin-angiotensin-aldosterone system and electrolyte imbalance. These conditions may be exacerbated after cardiac surgery, which may explain why CKD is an important risk factor for post-OP AF.

In our current analysis, smoking surprisingly emerged as a predictive factor with decreased odds. We hypothesise that smoking may serve as an indicator of the type of intervention. On one hand, smokers, who are at higher risk for developing atherosclerosis, were significantly more likely to undergo coronary artery bypass surgery. On the other hand, valvular heart disease—an established risk factor for AF as noted earlier—was considerably less common in these patients. This disparity may explain why these patients experienced fewer episodes of post-OP AF.

### Potential clinical applications and implications

Here, we present evidence that incorporating additional experimental data from isolated atrial cardiomyocytes can independently predict post-OP AF. This indicates that existing clinical data do not fully capture the atrial arrhythmogenic substrate in patients undergoing cardiac surgery, highlighting the need for including additional functional parameters in AF risk assessment. Specifically, abnormalities in Ca^2+^ handling may play a role in creating a substrate that predisposes patients to post-OP AF and is of potential interest for AF risk prediction.^1,24^ However, while isolation of atrial cardiomyocytes from patient samples and their *in vitro* investigation is in principle feasible, this technique is labor intensive, requires specialised equipment as well as a team of well-trained experts, precluding the direct translation of our findings into clinical practice. However, one could imagine several approaches to implement quantification of atrial cytosolic Ca^2+^ handling into clinical routine.

Direct quantification of Ca^2+^ transients in atrial trabeculae from right atrial biopsies could replace the complex isolation of atrial myocytes.^56^ Preliminary experiments using new Ca^2+^ fluorescent dyes in human atrial trabeculae are shown in Supplementary data online, *Video* and Supplementary data online, *Figure S4*.

Furthermore, since atrial Ca^2+^-handling abnormalities are also present in patients with paroxysmal and persistent AF and in patients with atrial cardiomyopathies in general, a non-invasive assessment of these values in patients may represent a promising technique to diagnose atrial cardiomyopathies and improve risk assessment in those patients. Recent work used tissue-doppler imaging of the LA together with simultaneous ECG recordings to assess electro-mechanical function.^57^ The authors found increased electro-mechanical dissociation in AF, and demonstrated that this is mainly due to impaired intracellular Ca^2+^ signalling.

Additionally, in the future, non-invasive quantification of atrial Ca^2+^ abnormalities may involve manganese-enhanced magnetic resonance imaging (MEMRI) of the atria. Recent clinical data suggest that MEMRI has exciting potential in the early detection of abnormalities in myocardial Ca^2+^ handling.^58^ Whether this approach can also be used in risk assessment and substratification of atrial cardiomyopathies remains open.

However, taken together, our data lay the foundation for establishing altered atrial Ca^2+^-handling abnormalities as an important parameter that holds promise to improve risk prediction and substratification of AF.

### Limitations

Further prolonging Holter-ECG recording after surgery or additional measures such as wearing smart watches before and after discharge could further improve the accuracy of AF detection. In addition, although we only included patients that had no documented AF before cardiac surgery, we cannot exclude that patients had silent AF episodes prior to cardiac surgery.^59^ However, according to recent studies, the clinical benefit of detecting silent AF episodes with continuous rhythm monitoring devices is unclear.^60–62^ Nevertheless, recent data from the CASTLE-AF and CASTLE-HTx trials suggest that reducing AF burden, rather than complete elimination of AF, is associated with a decrease in major adverse cardiovascular events.^63,64^ It would therefore be interesting to explore the potential of predicting AF burden using our novel predictive score.

Although our results demonstrate that the inclusion of additional Ca^2+^ handling parameters enhances the predictive accuracy of our model for the occurrence of post-OP AF in the immediate post-OP period, the performance of this model remains modest, with AUC values for the ROC curves below 0.8. However, we observed increases in predictive performance when including post-OP AF as an additional predictor for AF at discharge from 0.84 to 0.94, and for predicting AF during rehabilitation from 0.71 to 0.76 when including post-OP AF and AF at discharge as additional predictors (see Supplementary  *Text S4* and Supplementary data online, *Figure S5*). These findings indicate that the timing of rhythm status evaluation after cardiac surgery significantly influences the performance of post-OP AF prediction models. This temporal aspect should be carefully considered when comparing different post-OP AF prediction scores (see Supplementary data online, *Table S1*).

Furthermore, the modest accuracy of our model in predicting the occurrence of post-OP AF during the immediate post-OP period suggests that incorporating additional experimental or mechanistically relevant data could potentially further enhance predictive performance. When comparing the models for post-OP AF obtained from the dataset of clinical and single-cell parameters (*Figure 3A* and *B*) with the larger dataset of exclusively clinical parameters (*Figure 4C* and *D*), despite similarities of selected predictor variables and similar AUC values of ROC curves, caution must be taken due to the non-nested data structure resulting from exclusion of strongly unbalanced parameters in the smaller dataset of clinical and single-cell parameters (see Supplementary data online, *Figure S1*, see Methods for details). Our analysis already included blood-based biomarkers that are routinely determined in patients undergoing open heart surgery, including amongst others, potassium, Mg^2+^ levels, CRP and creatinine levels. However, we have not considered markers recently identified as independent predictors of AF, such as the N-terminal pro-B-type natriuretic peptide,^65,66^ high-sensitivity troponin,^67^ bone morphogenetic protein 10^66,68^ or angiopoietin 2.^66^ To the best of our knowledge, these markers are not directly linked to atrial Ca^2+^-handling abnormalities^41^ and we therefore assume that assessment of atrial cardiomyocyte Ca^2+^ handling may provide additional predictive values for the occurrence of AF. Specific micro-RNA have been suggested to directly modulate atrial Ca^2+^-handling and may therefore act as a more specific biomarker for atrial Ca^2+^-handling abnormalities. microRNA-106b-25 for example has been shown to regulate SaR Ca^2+^ release channels (RyR2) and microRNA-106b-25 levels were downregulated in patients with AF.^69^

## Conclusions

In this work, we present the first predictive post-OP AF risk model comprising not only clinical parameters but also experimental data from atrial cardiomyocytes isolated from right atrial appendages. Beyond established risk factors, our comprehensive analysis revealed that systolic cellular Ca^2+^ levels serve as an independent risk predictor for the development of post-OP AF. This emphasises the promising potential of adding detailed functional atrial parameters to better characterise pre-existing atrial cardiomyopathies, thereby improving the accuracy of predictive models for post-OP AF as well as late reoccurrences of AF after cardiac surgery. Consistent with this approach, previous studies have included atrial strain analysis as an additional predictor of post-OP AF risk.^24,70,71^ Moreover, ongoing advancements in the development of automated experimental methodologies, such as automated patch-clamp techniques and automated Ca^2+^ imaging, promise to facilitate the integration of the results of these experimental approaches into clinical studies and clinical risk prediction.^72^ This, in turn, could lead to the creation of novel diagnostic tools capable of more effectively predicting the development of AF in specific patient groups.

## Supplementary Material

ehaf609_Supplementary_Data

## Declarations

### Data Availability

All data are incorporated into the article and its online supplementary material.

### Pre-registered Clinical Trial Number

Not supplied.
