# Supplementary material for: Cytosolic calcium handling signature: integration with clinical predictors enhances prediction of post-operative atrial fibrillation
Source: Eur Heart J. 2025 Sep 9;47(13):1609–21. doi: 10.1093/eurheartj/ehaf609 (PMC13043196; doi:10.1093/eurheartj/ehaf609)
Supplement: ehaf609_Supplementary_Data [file ehaf609_supplementary_data.zip › Fakuade_EHJ-R2_Supplementary_Data_2025.pdf]

# - Supplemental Material -

Cytosolic calcium handling signature: integration with clinical predictors enhances prediction of post-operative atrial fibrillation

Funsho E. Fakuade, PhD[1,2,3,4*], Judith Gronwald, MD[1,2,5*], Paulina Brandes, MD[1,2], Yannic Döring[1,2], Tony Rubio, PhD[1,2], Fitzwilliam Seibertz, PhD[1,2,3], Maria Knierim, MD[2,4], Issam H. Abu-Taha, PhD[6], Aschraf El-Essawi, MD[2,4], Ahmad-Fawad Jebran, MD[2,4], Bernhard C. Danner, MD[2,4], Hassina Baraki, MD[2,4], Markus Kamler, MD[7], Ingo Kutschka, MD[2,4], Jordi Heijman, PhD[8,9], Dobromir Dobrev, MD[6,10,11], Constanze Schmidt, MD[2,5,12,13], Stefan M. Kallenberger, MD PhD[14,15,#], Niels Voigt, MD[1,2,3,#]

[1]Institute of Pharmacology and Toxicology, University Medical Centre Göttingen, Göttingen, Germany

[2]DZHK (German Centre for Cardiovascular Research), Partner Site Lower Saxony, Göttingen, Germany

[3]Cluster of Excellence "Multiscale Bioimaging: from Molecular Machines to Networks of Excitable Cells" (MBExC), University of Göttingen, Göttingen, Germany

[4]Department of Cardiothoracic and Vascular Surgery, University Medical Centre Göttingen, Göttingen, Germany

[5]Department of Cardiology and Pneumology, University Medical Centre Göttingen, Göttingen, Germany

[6]Institute of Pharmacology, West German Heart and Vascular Centre, University Duisburg-Essen, Essen, Germany

[7]Department of Thoracic and Cardiovascular Surgery, University Hospital Essen, Essen, Germany

[8]Gottfried Schatz Research Centre, Division of Medical Physics & Biophysics, Medical University of Graz, Graz, Austria.

[9]Department of Cardiology, Maastricht University Medical Centre and Cardiovascular Research Institute Maastricht, Maastricht University, Maastricht The Netherlands

[10]Department of Medicine and Research Centre, Montreal Heart Institute and Universitе́ de Montreal, Montreal, Canada

[11]Department of Integrative Physiology, Baylor College of Medicine, Houston, TX, USA

[12]Department of Cardiology, Heidelberg University Hospital, Heidelberg, Germany

[13]DZHK (German Centre for Cardiovascular Research), Partner Site Heidelberg/Mannheim, Heidelberg, Germany

[14]Health Data Science Unit, University Hospital Heidelberg and Centre for Quantitative Analysis of Molecular and Cellular Biosystems (BioQuant), University of Heidelberg, Heidelberg, Germany

[15]National Centre for Tumor Diseases, Department of Medical Oncology, Heidelberg University Hospital, Heidelberg, Germany

*FEF and JG contributed equally to this work

[#]SMK and NV share senior authorship

Running title: Ca^{2+} handling abnormalities and postoperative AF prediction

[#]Corresponding Authors:

Niels Voigt, Institute of Pharmacology and Toxicology, Robert-Koch-Straße 40, 37075 Göttingen, Germany, Tel.: 00495513965174, Fax: 0049 551 39 65169,
Email: niels.voigt@med.uni-goettingen.de
ORCID ID: 0000-0001-8230-2341

Stefan Kallenberger, BioQuant BQ054, University of Heidelberg, Im Neuenheimer Feld 267, 69120 Heidelberg, Germany, Tel.: 0049 6221 54 51308,
Email: stefan.kallenberger@bioquant.uni-heidelberg.de
ORCID ID: 0000-0003-3783-0246

Supplementary Methods

Supplementary Methods S1

Clinical data comprised demographic and basic medical history parameters [sex, age, BMI, height, smoking, heart rate before and after surgery], interventions [coronary artery bypass grafting (CABG), surgical aortic valve replacement (SAVR), mitral or tricuspid valve replacement/reconstruction], diseases [hypertension, diabetes, hyperlipidemia, heart failure New York Heart Association (NYHA) classification, stroke, transient ischemic attack (TIA), sleep apnea, chronic obstructive pulmonary disease (COPD), peripheral vascular disease, number of occluded coronary vessels, right coronary artery stenosis, history of myocardial infarction], laboratory values [creatinine pre-OP, hemoglobin post-OP, leucocytes post-OP, potassium pre- and post-OP, magnesium (Mg^{2+}) pre- and post-OP, reduced estimated glomerular filtration rate (eGFR) post-OP, C-reactive protein (CRP) post-OP], medication [angiotensin-converting-enzyme (ACE) inhibitor, angiotensin (AT1) receptor blocker, metoprolol, bisoprolol, Ca^{2+} antagonist, glucocorticoid, acetylsalicylic acid (ASS), diuretic, nitric oxide (NO) donor, lipid lowering drug, metformin, other oral antidiabetic drugs including insulin], echocardiographic parameters [diameters of the aortic root, left atrium (LA), the interventricular septum (IVSd), left posterior wall (LVPWd), the left ventricular end-diastolic diameter (LVEDD), left ventricular end systolic diameter (LVESD), left ventricular ejection fraction (LVEF), right ventricular diameter (RV), left ventricular hypertrophy (LVH), presence of an intra-cardiac shunt, post-OP pericardial effusion, degree of aortic stenosis (I-III°), degree of aortic valve insufficiency (AI) (I-III°), degree of mitral valve insufficiency (MI) (I-III°), degree of tricuspid valve insufficiency (TI) (I-III°), tricuspid annular plane systolic excursion (TAPSE), diastolic dysfunction, E/A ratio, E/e' ratio], details of the intervention [intervention time, bypass time, amount of heparin administered during intervention, post-OP Mg^{2+} substitution, post-OP potassium substitution] and anaesthesia [introduction of anaesthesia with etomidate, midazolam, propofol, sevoflouran or sufentanil, narcosis maintenance with midazolam, propofol or sevoflouran]. From intervention types, isolated mitral or tricuspid valve interventions were not used as predictors due to small patient numbers (n = 7 with mitral, n = 2 with tricuspid valve intervention).

Supplementary Methods S2

For calcium measurements in isolated human trabeculae carneae, a dye loading procedure adapted from a protocol published by Borysova et al.[1] was used. Right and left atrial appendages (RAA & LAA) were collected from patients with sinus rhythm undergoing open heart surgery and transported to our laboratories in cardioplegic solution (100 mmol/L NaCl, 10 mmol/L KCl, 5 mmol/L $MgSO_4$, 1.2 mmol/L KH_2PO_4, 30 mmol/L 2,3 butane dione monoxime

[BDM], 50 mmol/L Taurin, 20 mmol/L glucose, and 5 mmol/L 3-(N-morpholino) propanesulfonic acid; pH 7.0). Trabeculae carneae were isolated and incubated in continuously aerated Tyrode's solution for 30 min to remove residual BDM. In parallel, a 1 mmol/L solution of Cal-590 AM and 100 mg/mL pluronic acid in DMSO was prepared as previously described.[1] 5 µL of this solution was mixed with 1 mL of Tyrode's solution (120 mmol/L NaCl, 1 mmol/L $MgCl_2$, 0.2-4 mmol/L $CaCl_2$, 5.4 mmol/L KCl, 22.6 mmol/L $NaHCO_3$, 4.2 mmol/L NaH_2PO_4, and 5.6 mmol/L glucose; pH 7.4 was maintained by gassing with 5 % CO_2/95 % O_2). The trabeculae were placed in this solution for 15 min and then transferred into continuously aerated Tyrode's solution for 20 min to facilitate de-esterification. To prevent motion artefacts, trabeculae were incubated with 5 µmol/L Blebbistatin in continuously aerated Tyrode's solution for 15 min directly before the recording. Afterwards, the trabeculae were transferred into a measuring chamber and fixed in place with insect pins without piercing them.

The trabeculae were electrically point-stimulated using a custom-built platinum/iridium bipolar electrode at stimulation frequencies between 0.25 and 3 Hz. The calcium indicator was excited at wavelengths ranging from 475 nm to 575 nm using an LED (LEX3 illumination system, Brainvision/Sci-Media). Emission was measured between 560 nm and 590 nm using a complementary Metal-Oxide semiconductor (CMOS) camera (Micam Ultima 10 x 10 mm^2 sensor, Brainvision/Sci-Media). MappingLab's OMapScope software version 5.9.41, kindly provided by them, was used for the analysis of the data. The time constant (τ) of the decay of the calcium transient (CaT) was automatically calculated by the software. As a relative measure for systolic Ca^{2+} levels, the maximum change in fluorescence intensity relative to the minimal fluorescence intensity ($\Delta F/F0$) was determined. **Supplementary Video S1** shows an exemplary recording of a stimulated calcium signal.

Supplementary Text

Supplementary Text S1

The robustness of single-cell measurements was assessed by comparing the uncertainty of measurements in single patients to the variability of values between patients. Of the 71 patients, in 18 patients only one cell was measured, in 11 patients two cells. The mean and median cell number per patient was $\bar{n} = 3$. The distribution of the number of cardiomyocytes per patient is shown in Supplementary **Fig. S2A**.

To estimate the error of single-cell data for the 18 cases with only one measured cell per patient, we fitted linear error models to standard errors from j patients in whom $n \geq 3$ cells were measured for the $i = 1 \dots 9$ variables. The linear error model for each variable i

$$\varepsilon_i(y_i) = m_{1,i} y_{i,j} + m_{2,i} \max(y_{i,j})$$

contained the coefficient $m_{1,i}$ multiplied by variable values for different patients and the coefficient $m_{2,i}$ multiplied by the maximal value of the variable (centre panels in **Fig. S2B** to **D**). Parameters were estimated using the least absolute residual algorithm as implemented in the MATLAB function 'fit'. The start point was $m_{1,i,0} = m_{2,i,0} = 0.1$. The error model was then used to estimate the error of measurements from patients with $n = 1$ cell to assess the robustness of single-cell measurements. Results are indicated exemplarily for the three exemplary variables that significantly differed between post-OP AF and SR groups, '$[Ca^{2+}]_{syst.}$,' '$[Ca^{2+}]_i$ amplitude,' and 'CaT decay time' (**Fig. S2B** to **D**).

The right column of **Fig. S2B** to **D** indicates the standard error of all patients normalised by the overall mean value (blue dashed line), indicating variability of measurements between patients. It is shown together with SEM values from patients with cells (red circles), indicating the uncertainty of measurements in patients, in whom more than one cardiomyocyte was measured, normalised by means. Further, estimated SE values are indicated for patients with cells normalised by the respective parameter value (yellow circles). In all parameters, the SE values describing the parameter variability between patients were, in most cases, larger than uncertainties of measurements in single patients (SEM values in patients with $n \geq 2$ cells and SE estimates in patients with $n = 1$ cell). Therefore, we conclude that measurements of patients with one or two cells were sufficiently robust to include them in the analysis.

Supplementary Text S2

The score was tested in an independent dataset provided by the group of Prof D. Dobrev.[2] The external dataset contained complete measurements of systolic Ca^{2+} concentrations together

with the selected clinical parameters (age, LA diameter, post-OP Mg^{2+} serum concentration, reduced eGFR post-OP, smoking status, aortic valve stenosis degree, tricuspid valve insufficiency degree). These data were fully available in a total of n=23 patients.

Due to differences in the study design, study population, and differences in data collection, the following assumptions were considered:

A. Smoker status: The study by Heijman et al.[2] collected whether a patient was non-smoker (n=12), active smoker (n=6), or ex-smoker (n=5). In this study, only the classes 'smoker' and 'ex-smoker' were documented. We therefore tested predictive performance when (A1) including ex-smokers in the class 'non-smoker' or (A2) including ex-smokers in the class 'smoker'.

B. Degree of aortic valve stenosis (AVS): Different from this work, in the study by Heijman et al.,[2] patients were included who underwent prior surgical AVS treatment. Besides three degrees of aortic valve stenosis, the status 'after AVS surgical treatment' was documented. This was the case for n=12 patients (of the remaining 11 patients, 7 patients were not diagnosed with AVS, one patient with AVS grade I, one patient with AVS grade II, and 2 patients with AVS grade III). In our study, however, only the classes 'no AVS' or degrees I to III (associated with values [0,1,2,3]) were documented. We therefore tested the predictive performance when (B1) including the class 'status after surgical AVS treatment' in the class 'severe AVS / grade III' or (B2) including the class 'status after surgical AVS treatment' in the class 'no AVS'.

C. Differences between patient groups. In the dataset of Heijman et al.,[2] patients were of significantly older and had a higher degree of AVS. Further, in the dataset of Heijman et al.,[2] higher systolic $[Ca^{2+}]_i$ and post-OP Mg^{2+} concentrations were measured. Besides differences between patient groups, we expect batch effects in experimental measurements possibly caused by differences in sample handling, clinical laboratories, and quantification devices. The average values are listed in the following table:

Supplementary Context Table 1.
Characteristics of classification parameters in original and validation datasets.

	This study	Heijman et al.[2]	Factor
$[Ca^{2+}]_i$ in nmol/l, systolic	427.7	601.6	1.41*
Age in years	65.5	72.3	1.12*
LA diameter in mm	41.5	41.2	0.99
Mg^{2+} post OP in mmol/l	0.805	1.279	1.59***
Fraction with reduced eGFR post OP (eGFR < 60ml/min/1,73 m^2)	0.197	0.391	1.98
Degree of aortic valve stenosis (I-III°)	0.521	1.957	3.75***
Fraction of nicotine abuse/history of smoking	0.423	0.261	0.62
Degree of tricuspid valve insufficiency (I-III°)	0.662	0.565	0.85

*p<0.05; ***p<0.001 of Wilcoxon rank sum test

In the table, the assumptions 'A1' (inclusion of ex-smokers in the class 'non-smokers') and 'B1' (inclusion of patients 'after surgical AVS treatment' in the class 'AVS degree III' / severe) were made. Due to significant differences between groups, particularly in patient age, the post-OP Mg^{2+} concentration, the fraction of patients with reduced eGFR and the degree of AVS, we normalised values by the indicated factors to correct for systematic differences between groups.

To assess the validity of the derived model for post-OP AF prediction in the dataset by Heijman et al.,[2] we tested the model given possible combinations of assumptions (A1, A2, B1, B2) for adaptations between datasets (**Supplementary Context Table 2**).

Supplementary Context Table 2.
AUC values of model for post-OP AF prediction applied on external dataset.

Assumptions for adaptation between datasets				Model including $[Ca^{2+}]_i$ systolic AUC values, 95% CI*	Model without Ca^{2+} parameter AUC values, 95% CI
A1 Inclusion of ex-smokers in non-smokers	A2 Inclusion of ex-smokers in smokers	B1 Inclusion of 'post AVS treatment' in 'severe AVS'	B2 Inclusion of 'post AVS treatment' in 'no AVS'		
+	–	+	–	0.79 [0.54, 0.95]	0.76 [0.52, 0.92]
+	–	–	+	0.69 [0.44, 0.87]	0.61 [0.28, 0.82]
–	+	+	–	0.72 [0.46, 0.91]	0.69 [0.42, 0.89]
–	+	–	+	0.62 [0.36, 0.85]	0.60 [0.32, 0.81]

* 95% confidence intervals (CI) were estimated by bootstrapping with 1000 samples.

Essentially, the AUC values for post-OP AF prediction obtained when applying the models derived from the dataset of this study on the external dataset by Heijman et al.[2] were in all cases larger when the single-cell parameter '$[Ca^{2+}]_i$ systolic' was additionally included. The largest AUC was obtained when values of ex-smokers were included in the class 'non-smokers' (A1) and values of patients 'after surgical AVS treatment' were included in the class 'severe AVS.' The latter assumption (B1) implies irreversible remodelling of the atria that persists even after surgical AVS treatment. We conclude that in the external dataset, similarly as in the dataset of the present study, including the single-cell Ca^{2+} transient parameter improves the prediction of post-OP AF.

Supplementary Text S3

Differences in NYHA heart failure classification, the fraction of reduced eGFR and the fraction of smokers were smaller between AF and SR groups at the time of discharge and during rehabilitation treatment. Further, the age difference between AF and SR groups decreased from the time of hospital discharge to the time of a rehabilitation treatment. Contrarily, in patients with AF at discharge or with AF during rehabilitation treatment, higher severity levels of AVS, according to degrees I to III, were observed again indicating subgroups with different pathologies (**Supplementary Fig. S4**).

Comparing clinical characteristics of patients with post-OP AF, AF at discharge and AF at rehabilitation indicated an over-representation of patients with AVS in the group of patients with AF at discharge and AF at rehabilitation (**Supplementary Table S8**). In patients with AF at discharge, the percentage of 'aortic valve replacement' and 'AVS degree' were significantly higher. Further, the interventricular septum ('IVSd') and LVPWd were larger, which can be associated with AVS. Further, in patients with AF at discharge, relative to patients with post-OP AF, the percentage of AT1 blocker intake was higher and the percentage of ACE inhibitors lower. A parallel might be drawn to a study in patients undergoing coronary artery bypass grafting surgery showing that in patients treated by ACE inhibition, opposed to AT1 blockade, intraoperative fibrinolysis was increased and the length of the hospital stay was shorter.[3]

Supplementary Text S4

We observed that AF was more reliably predicted at the time of discharge, as indicated by an AUC value of 0.84 (**Supplementary Fig. S5A**), followed by prediction of post-OP AF within the in-hospital post-OP period (AUC=0.69) and AF during rehabilitation treatment (AUC=0.71). Next, we included the information of post-OP during the in-hospital post-OP period for the prediction of AF at discharge, which resulted in a further increased AUC value of 0.94 (**Supplementary Fig. S5B**). When including post-OP and AF at discharge as predictors for AF during rehabilitation treatment, the AUC value increased to 0.76.

The most predictive parameters for AF at discharge, associated with increased AF risk, were the post-OP heart rate, age, aortic valve replacement, BMI and AT1 blocker intake (**Supplementary Fig. S5C**). For example, aortic valve replacement was associated with 4.9-fold higher odds for AF at discharge. History of one or more myocardial infarctions was predictive for decreased risk, again indicating differences in patient groups undergoing cardiac surgery. Parameters predictive for AF during rehabilitation treatment, obtained from sequential

feature selection, included similar factors as those identified for post-OP AF prediction, including the degree of AVS, LA diameter and smoking, as well as the post-OP heart rate as in the case of predicting AF at discharge, additionally including presence of a post-OP pericardial effusion (PE, **Supplementary Fig. S5D**).

Including AF at earlier time points as predictors resulted in slightly changed sets of selected parameters (**Supplementary Figs. S5E** and **F**). Post-OP AF was highly predictive for AF at discharge (**Supplementary Fig. S5E**) – because all subjects with AF at discharge had post-OP AF. For this reason, the model parameter (odds ratio) for this predictor was not identifiable. For predicting AF during rehabilitation treatment, AF at discharge, post-OP AF, degree of aortic valve stenosis, smoking and presence of a post-OP PE were selected (**Supplementary Fig. S5F**).

Supplementary Tables

Supplementary Table S1. Comparison of various score models for post-OP AF prediction

		Burgos *et al.* 2021, Ann Card Anaesth	Lin *et al.* 2018, Heart Surg Forum	Mariscalco *et al.* 2014, J Am Heart Assoc	Tran *et al.* 2015, J Cardiothorac Vasc Anesth	El-Chami *et al.* 2012, Am J Cardiol	Magee *et al.* 2007, Ann Thorac Surg	Amar *et al.* 2004, J Am Coll Cardiol	Mathew *et al.* 2004, JAMA	Zaman *et al.* 2000, Circulation	post-OP AF (Fig. 1)	post-OP AF + Ca^{2+}-parameters (Fig. 2)
General	AUC of ROC curves	0.78	0.60	0.71	0.68	0.68	0.72	0.69	0.77	N/A	0.69	0.72
	Number of patients	3113	1307	3113	999	19895	19083	1851	4657	326	530	71
Commonly identified risk factors	Age	1	1	1	1	1	1	1	1	1	1	1
	Heart Failure	1		1			1	1				
	Weight					1	1					
	Height					1	1					
	LA diameter		1		1						1	1
	Reduced eGFR		1	1							1	1
	Valvular Heart disease			1	1				1		1	1
	COPD/Lung disease			1			1		1			
	White race		1				1					
Sex	Female sex	1										
	Male sex									1		
Diseases	Previous stroke	1										
	Hypertension	1										
	Diabetes	1										
	Peripheral vascular disease					1						
	Previous CABG						1					
	Pre-OP arrhythmia						1					
	History of AF							1	1			
	p-wave duration							1		1		
Surgery related	Increased post-OP $[Mg^{2+}]_{Serum}$										1	1
	On-pump						1					
	Prolonged ventilator usage						1					
	Emergency surgery			1								
	Pre-OP intra-aortic balloon pump			1								
Drug and substance intake	Glucocorticoid treatment										1	
	ACE-inhibitor treatment						1		1			
	Withdrawal of ACE-inhibitors								1			
	β-Blocker treatment						1		1			
	Withdrawal of β-blockers								1			
	Anticoagulant treatment						1					
	Smoking						1				-1	-1
	NSAR								1			
	K^+-supplementation								1			
	Systolic Ca^{2+}											-1

N/A, data not available

Supplementary Table S2. Comparisons of clinical parameters between group of patients with available single-cell calcium measurements (n=71) and the group of patients with known post-OP AF status without available single-cell calcium measurements (n=469).

	Patients with single-cell calcium measurements, (n=71)	Patients with known post-OP AF status without single-cell calcium measurements (n=469)	*p*-value
Post-OP AF	19 (26.8%), n=71	154 (33.6%), n=459	0.28
Age (y)	65.5 ± 12, n=71	66 ± 10.1, n=459	0.92
Sex	61 (85.9%), n=71	382 (83.2%), n=459	0.73
Height (m)	1.76 ± 0.079, n=71	1.74 ± 0.0802, n=456	0.059
Body mass index (kg/m²)	27.7 ± 4.28, n=71	28.3 ± 4.69, n=455	0.37
Coronary artery bypass grafting	57 (80.3%), n=71	381 (83.2%), n=458	0.61
Aortic valve replacement	20 (28.2%), n=71	125 (27.3%), n=458	0.89
Intervention time (min)	278 ± 80.5, n=66	277 ± 151, n=430	0.69
Intervention time (min)	127 ± 42.3, n=62	125 ± 46, n=400	0.82
Heparin (IE) during intervention	30.4 ± 7.78, n=51	29.6 ± 7.41, n=378	0.68
Narcosis initiation, midazolam	41 (68.3%), n=60	259 (71.5%), n=362	0.65
Narcosis initiation, sevoflouran	16 (26.7%), n=60	81 (22.4%), n=362	0.51
Narcosis initiation, sufentanil	16 (26.7%), n=60	80 (22.1%), n=362	0.41
Narc. maintenance, propofol	15 (23.1%), n=65	98 (25.6%), n=383	0.76
Arterial hypertension	59 (83.1%), n=71	407 (88.9%), n=458	0.17
Diabetes	17 (23.9%), n=71	152 (33.3%), n=456	0.13
Hyperlipidemia	35 (49.3%), n=71	262 (57.2%), n=458	0.25
Smoking	30 (42.3%), n=71	186 (40.8%), n=456	0.9
NYHA classification	2.2 ± 0.717, n=64	2.3 ± 0.806, n=401	0.29
COPD	12 (17.1%), n=70	52 (11.4%), n=457	0.17
Peripheral vascular disease	13 (18.6%), n=70	164 (22.5%), n=454	0.54
Heart rate pre-OP	68.4 ± 12.8, n=67	69 ± 12, n=438	0.42
Heart rate post-OP	73 ± 10, n=64	74.7 ± 11.5, n=440	0.25
Creatinine pre-OP (µmol/L)	0.999 ± 0.275, n=71	1.05 ± 0.532, n=459	0.52
Hemoglobin post-OP (g/dL)	10.2 ± 1.02, n=70	10.3 ± 1.25, n=459	0.54
Leucocytes post-OP (10^3/µL)	10.5 ± 3.24, n=70	10.7 ± 3.48, n=459	0.79
K^+ pre-OP (mmol/L)	4.19 ± 0.396, n=67	4.13 ± 0.403, n=435	0.26
K^+ post-OP (mmol/L)	4.15 ± 0.298, n=68	4.24 ± 0.337, n=450 *	0.024
Mg^{2+} pre-OP (mmol/L)	0.923 ± 0.195, n=59	0.967 ± 0.207, n=407	0.12
Mg^{2+} post-OP (mmol/L)	0.814 ± 0.119, n=58	0.856 ± 0.175, n=401	0.11
Reduced eGFR post-OP	14 (20.6%), n=68	89 (19.6%), n=455	0.87
C-reactive protein post OP (mg/L)	93.2 ± 43.3, n=69	78.5 ± 42.9, n=457 **	0.0029
N stenosed vessels	2.56 ± 0.906, n=71	2.55 ± 0.889, n=454	0.7
RCA stenosis ≥70%	46 (64.8%), n=71	282 (63.5%), n=444	0.89
Previous myocardial infarction	0.352 ± 0.588, n=71	0.457 ± 0.567, n=442	0.075
Aortic root (mm)	33.9 ± 5.89, n=67	33.2 ± 4.78, n=385	0.82
LA diameter (mm)	41.6 ± 6.59, n=70	41.6 ± 5.96, n=417	0.75
IVSd (mm)	12.8 ± 2.69, n=71	12.6 ± 2.41, n=428	0.33
LVPWd (mm)	12.1 ± 2.34, n=69	11.9 ± 2.34, n=410	0.3
LVEDD (mm)	49 ± 7.66, n=71	49.2 ± 7.43, n=430	0.79
LVESD (mm)	32.3 ± 6.38, n=63	33.5 ± 20.3, n=336	0.82
LVEF (%)	51.1 ± 11.5, n=71	52 ± 11.6, n=451	0.46
LVH	57 (80.3%), n=71	340 (76.9%), n=442	0.65
Right ventricular diameter (mm)	37.2 ± 5.29, n=50	36.1 ± 5.2, n=287	0.13
Pericardial effusion post OP	28 (42.4%), n=66	167 (38.7%), n=431	0.59
Tricuspid valve insufficiency (I-III°)	0.662 ± 0.584, n=71	0.706 ± 0.602, n=456	0.57
Aortic stenosis (I-III°)	0.521 ± 1.03, n=71	0.666 ± 1.17, n=455	0.46
Aortic valve insufficiency (I-III°)	0.592 ± 0.871, n=71	0.595 ± 0.811, n=457	0.73
Mitral valve insufficiency (I-III°)	0.873 ± 0.675, n=71	0.978 ± 0.698, n=456	0.24

TAPSE (mm)	20 ± 5.92, n=57	21.5 ± 6.08, n=354 *	0.046
Diastolic dysfunction	1.06 ± 0.624, n=62	0.937 ± 0.722, n=395	0.15
E/A ratio	1.09 ± 0.486, n=55	1.13 ± 0.469, n=305	0.2
E/e ratio	9.16 ± 4.28, n=48	9.33 ± 3.73, n=275	0.67
ACE inhibitor	36 (51.4%), n=70	222 (48.5%), n=455	0.7
AT1 blocker	19 (27.1%), n=70	125 (27.5%), n=454	1
Metoprolol	12 (16.9%), n=71	79 (17.2%), n=459	1
Bisoprolol	29 (40.8%), n=71	176 (38.3%), n=459	0.7
Calcium antagonist	18 (25.4%), n=71	125 (27.5%), n=455	0.78
Diuretic drug	25 (35.2%), n=71	162 (35.6%), n=455	1
Lipid lowering drug	52 (73.2%), n=71	358 (78.7%), n=455	0.36
ASS	47 (66.2%), n=71	332 (72.3%), n=459	0.32
Anti-diabetic drug other than metformin, incl. insulin	10 (14.3%), n=70	78 (16.8%), n=454	0.73
Mg^{2+} substitution	59 (83.1%), n=71	383 (83.4%), n=459	1

For continuous parameters and ordinal parameters with more than two levels, means and standard deviations are given, for binary parameters, total counts and percentages are indicated; **p*<0.05, ***p*<0.01 versus SR from Wilcoxon rank sum test for continuous variables and from Fisher exact test for categorical variables. ACE – angiotensin converting enzyme, ASS – acetylsalicylic acid, AT1 – angiotensin receptor 1, COPD – chronic obstructive pulmonary disease, eGFR – estimated glomerular filtration rate, IVSd – interventricular septum, LA – left atrium, LVEDD – left ventricular end-diastolic diameter, LVEF – left ventricular ejection fraction, LVESD – left ventricular end-systolic diameter, LVH – left ventricular hypertrophy, LVPWd – left posterior wall, NYHA – New York Heart Association, TAPSE – tricuspid annular plane systolic excursion.

Supplementary Table S3. Comparisons of Ca^{2+} measurements between post-OP SR and AF.

	**Post-OP SR** (n=52)	**Post-OP AF** (n=19)	p-value
C_m in pF	87.5 ± 34	91 ± 27.8	0.69
$[Ca^{2+}]_i$ in nmol/L, diastolic	222 ± 123	200 ± 75.4	0.47
$[Ca^{2+}]_i$ in nmol/L, systolic	455 ± 209	353 ± 109 *	0.048
$[Ca^{2+}]_i$, amplitude in nmol/L	233 ± 133	153 ± 62.6 *	0.015
CaT decay time in ms	414 ± 129	505 ± 185 *	0.024
CaT time to peak in ms	85.9 ± 23.2	89 ± 18.9	0.60
$I_{Ca,L}$ peak in pA	550 ± 308	524 ± 228	0.73
$I_{Ca,L}/C_m$ in pA/pF	6.65 ± 3.03	6.38 ± 2.63	0.74
Integral of $I_{Ca,L}$ in pA·ms	$7.38 \cdot 10^3 \pm 4.03 \cdot 10^3$	$7.26 \cdot 10^3 \pm 3.23 \cdot 10^3$	0.91

Means and standard deviations from n=71 subjects are given, C_m, membrane capacitance; $[Ca^{2+}]$, calcium concentration, $I_{Ca,L}$, calcium current; *p<0.05 versus SR from one-way ANOVA rank sum test.

Supplementary Table S4. Predicting postoperative AF from clinical parameters single-cell calcium measurements.

	**Postoperative AF vs. SR**			
	Coefficient (95% CI)	Odds ratio (95% CI)	Variable increment	p-value
$[Ca^{2+}]_{syst}$ (nmol/l)	-0.577 (-1.167, 0.013)	0.561 (0.311, 1.013)	100 nmol/L	0.0552
Age (y)	0.931 (0.165, 1.698)	2.538 (1.179, 5.463)	10 years	0.0172
Aortic stenosis (I-III°)	0.450 (-0.209, 1.109)	1.569 (0.811, 3.033)	degree	0.181
Nicotine	-1.081 (-2.679, 0.517)	0.339 (0.069, 1.677)		0.185
Reduced post-OP. eGFR	1.252 (-0.605, 3.110)	3.498 (0.546, 22.418)		0.186
LA diameter (mm)	0.219 (-0.291, 0.729)	1.245 (0.747, 2.072)	5 mm	0.400
Tricuspid valve insufficiency (I-III°)	0.165 (-1.086, 1.415)	1.179 (0.338, 4.116)	degree	0.796
Mg^{2+} post-OP (mmol/L)	0.073 (-0.546, 0.693)	1.076 (0.579, 1.999)	0.1 mmol/L	0.816
Intercept	-7.856 (-17.131, 1.419)			0.0969

$[Ca^{2+}]_{syst}$, systolic calcium concentration, eGFR, estimated glomerular filtration rate; LA, left atrial. Model parameters were ordered according to p-values, numeric parameters were scaled to representative variable increments as indicated in the fourth column.

Supplementary Table S5. Comparisons of clinical parameters between post-OP SR and AF groups.

	**Post-OP SR**, n=357	**Post-OP AF**, n=173 (32.6%)	*p*-value
Age	64.3 ± 10.6, n=357	69.3 ± 8.99, n=173 ***	$1.5 \cdot 10^{-7}$
Male gender	305 (85.4%), n=357	138 (79.8%), n=173	0.11
Height (m)	1.74 ± 0.0789, n=354	1.74 ± 0.0831, n=173	0.52
Body mass index (kg/m^2)	28.2 ± 4.71, n=353	28.2 ± 4.5, n=173	0.75
Coronary artery bypass grafting	294 (82.6%), n=356	144 (83.2%), n=173	0.9
Aortic valve replacement	96 (27.0%), n=356	49 (28.3%), n=173	0.76
Intervention time (min)	270 ± 67.1, n=334	290 ± 231, n=162	0.94
Bypass time (min)	124 ± 40.4, n=311	128 ± 54.7, n=151	0.85
Heparin (IE) during intervention	29.5 ± 7.56, n=296	30.1 ± 7.2, n=133	0.47
Narcosis initiation, etomidate	11 (3.9%), n=283	4 (2.9%), n=139	0.78
Narcosis initiation, midazolam	204 (72.1%), n=283	96 (69.1%), n=139	0.57
Narcosis initiation, propofol	5 (1.8%), n=283	4 (2.9%), n=139	0.48
Narcosis initiation, sevoflouran	65 (23.0%), n=283	32 (23.0%), n=139	1
Narcosis initiation, sufentanil	64 (22.6%), n=283	32 (23.0%), n=139	1
Narc. maintenance, midazolam	50 (16.4%), n=305	19 (13.3%), n=143	0.48
Narc. maintenance, propofol	73 (23.9%), n=305	40 (28.0%), n=143	0.41
Narc. maintenance, sevoflouran	301 (98.7%), n=305	141 (98.6%), n=143	1
Intracardiac device	15 (4.4%), n=339	14 (8.6%), n=162	0.067
Arterial hypertension	312 (87.6%), n=356	154 (89.0%), n=173	0.77
Diabetes mellitus	109 (30.6%), n=356	60 (35.1%), n=171	0.32
Hyperlipidemia	203 (57.0%), n=356	94 (54.3%), n=173	0.58
Smoking	161 (45.4%), n=355	55 (32.0%), n=172 **	0.0035
NYHA classification	2.21 ± 0.84, n=309	2.43 ± 0.673, n=156 **	0.0083
Stroke	28 (7.9%), n=356	15 (8.8%), n=171	0.74
Transient ischemic attack	11 (3.1%), n=355	8 (3.5%), n=171	0.8
Sleep apnea	25 (7.1%), n=354	12 (7.1%), n=168	1
COPD	40 (11.3%), n=355	24 (14.0%), n=172	0.39
Peripheral vascular disease	137 (21.2%), n=354	40 (23.5%), n=170	0.57
Heart rate pre-OP	68.7 ± 12.3, n=343	69.4 ± 11.6, n=162	0.68
Heart rate post-OP	74.4 ± 10.5, n=347	74.8 ± 13, n=157	0.75
Creatinine pre-OP (µmol/L)	1.03 ± 0.473, n=357	1.07 ± 0.568, n=173	0.67
Hemoglobin post-OP (g/dL)	10.3 ± 1.25, n=356	10.3 ± 1.17, n=173	1
Leucocytes post-OP (10^3/µL)	10.7 ± 3.58, n=356	10.6 ± 3.16, n=173	0.97
K^+ pre-OP (mmol/L)	4.14 ± 0.399, n=335	4.14 ± 0.408, n=167	0.97
K^+ post-OP (mmol/L)	4.2 ± 0.336, n=350	4.29 ± 0.322, n=168 **	0.003
Mg^{2+} pre OP (mmol/L)	0.958 ± 0.202, n=313	0.968 ± 0.213, n=153	0.78
Mg^{2+} post-OP (mmol/L)	0.832 ± 0.141, n=304	0.888 ± 0.211, n=155 **	0.0037
Reduced eGFR post-OP	54 (15.3%), n=352	49 (28.7%), n=171 ***	0.00062
C-reactive protein post-OP (mg/L)	81.9 ± 44.6, n=353	77.4 ± 40.1, n=173	0.44
N stenosed vessels	2.58 ± 0.879, n=353	2.5 ± 0.914, n=172	0.22
RCA stenosis ≥ 70%	225 (64.7%), n=348	103 (61.7%), n=167	0.56
Previous myocardial infarction	0.454 ± 0.588, n=350	0.417 ± 0.531, n=163	0.64
Aortic root (mm)	33.3 ± 4.87, n=303	33.5 ± 5.14, n=149	0.83
LA diameter (mm)	41 ± 5.73, n=323	42.8 ± 6.48, n=164 *	0.011
IVSd (mm)	12.6 ± 2.45, n=338	12.6 ± 2.46, n=161	0.98
LVPWd (mm)	12 ± 2.37, n=323	11.9 ± 2.28, n=156	0.6
LVEDD (mm)	49 ± 6.9, n=335	49.5 ± 8.48, n=166	0.59
LVESD (mm)	33.7 ± 22.6, n=259	32.5 ± 7.85, n=140	0.74
LVEF (%)	52.7 ± 11.1, n=352	50.2 ± 12.3, n=170 *	0.026
LVH	267 (76.9%), n=347	130 (78.3%), n=166	0.82
Right ventricular diameter (mm)	36.2 ± 5.11, n=229	36.4 ± 5.46, n=108	0.62
intra-cardiac shunt	27 (8.9%), n=305	11 (7.2%), n=152	0.6
Pericardial effusion post-OP	124 (36.9%), n=336	71 (44.1%), n=161	0.14
Tricuspid valve insufficiency (I-III°)	0.656 ± 0.601, n=355	0.791 ± 0.585, n=172 **	0.0098l

Aortic valve stenosis (I-III°)	0.576 ± 1.09, n=354	0.791 ± 1.25, n=172	0.059
Aortic valve insufficiency (I-III°)	0.587 ± 0.812, n=356	0.61 ± 0.834, n=172	0.83
Mitral valve insufficiency (I-III°)	0.916 ± 0.679, n=356	1.06 ± 0.721, n=171 *	0.037
TAPSE (mm)	21 ± 5.94, n=277	21.9 ± 6.31, n=134	0.27
Diastolic dysfunction	0.961 ± 0.719, n=310	0.939 ± 0.695, n=147	0.85
E/A ratio	1.11 ± 0.471, n=246	1.15 ± 0.471, n=114	0.67
E/e' ratio	9.16 ± 3.86, n=234	9.68 ± 3.69, n=89	0.2
ACE inhibitor	176 (49.9%), n=353	82 (46.8%), n=172	0.52
AT1 blocker	102 (28.9%), n=353	42 (24.6%), n=171	0.35
Metoprolol	59 (16.5%), n=357	32 (18.5%), n=173	0.62
Bisoprolol	137 (38.4%), n=357	68 (39.3%), n=173	0.85
Nebivolol	11 (3.1%), n=357	10 (5.8%), n=173	0.16
Other beta blocker	11 (3.1%), n=357	7 (4.0%), n=173	0.61
Calcium antagonist	87 (24.6%), n=354	56 (32.6%), n=172	0.06
Diuretic drug	112 (31.6%), n=354	75 (43.6%), n=172 **	0.0087
Nitrate drug	42 (11.9%), n=354	19 (11.0%), n=172	0.88
Lipid lowering drug	280 (79.1%), n=354	130 (75.6%), n=172	0.37
ASS	255 (71.4%), n=357	124 (71.7%), n=173	1
Clopidogrel	33 (9.2%), n=357	11 (6.4%), n=173	0.31
Other aggregation inhibitor	19 (5.3%), n=357	7 (4.0%), n=173	0.67
Other anti-diabetic drug, incl. insulin	51 (14.4%), n=353	37 (20.6%), n=171	0.079
Metformin	49 (13.9%), n=353	30 (17.5%), n=171	0.3
Glucocorticoid	10 (2.8%), n=353	14 (8.2%), n=171 *	0.012
Mg^{2+} substitution	295 (82.6%), n=357	147 (85.0%), n=173	0.54
K^{+} substitution	324 (90.8%), n=357	161 (93.1%), n=173	0.41

For continuous parameters and ordinal parameters with more than two levels, means and standard deviations are given, for binary parameters, total counts and percentages are indicated; **p*<0.05, ***p*<0.01, ****p*<0.001 versus SR from Wilcoxon rank sum test for continuous variables and from Fisher exact test for categorical variables. ACE – angiotensin converting enzyme, ASS – acetylsalicylic acid, AT1 – angiotensin receptor 1, COPD – chronic obstructive pulmonary disease, eGFR – estimated glomerular filtration rate, IVSd – interventricular septum, LA – left atrium, LVEDD – left ventricular end-diastolic diameter, LVEF – left ventricular ejection fraction, LVESD – left ventricular end-systolic diameter, LVH – left ventricular hypertrophy, LVPWd – left posterior wall, NYHA – New York Heart Association, TAPSE – tricuspid annular plane systolic excursion

Supplementary Table S6. Comparisons of clinical parameters between at discharge SR and AF groups.

	**SR at discharge**, n=487	**AF at discharge**, n=21 (4.1%)	*p*-value
Post-OP AF	134 (27.8%), n=482	21 (100%), n=21 ***	$6.8 \cdot 10^{-12}$
Age	65.5 ± 10.5, n=487	71.4 ± 7.57, n=21 **	0.0093
Male gender	409 (84.0%), n=487	17 (81.0%), n=21	0.76
Height (m)	1.74 ± 0.0806, n=484	1.73 ± 0.0873, n=21	0.52
Body mass index (kg/m^2)	28.1 ± 4.62, n=483	29.8 ± 4.98, n=21	0.07
Coronary artery bypass grafting	403 (82.9%), n=486	17 (81.0%), n=21	0.77
Aortic valve replacement	129 (26.5%), n=486	11 (52.4%), n=21 *	0.021
Intervention time (min)	275 ± 145, n=454	256 ± 72.7, n=20	0.3
Bypass time (min)	124 ± 40.9, n=422	112 ± 35.2, n=17	0.27
Heparin (IE) during intervention	29.7 ± 7.49, n=393	29 ± 4.76, n=17	0.86
Narcosis initiation, etomidate	15 (3.8%), n=390	0 (0.0%), n=16	1
Narcosis initiation, midazolam	276 (70.8%), n=390	14 (87.5%), n=16	0.26
Narcosis initiation, propofol	9 (2.3%), n=390	0 (0.0%), n=16	1
Narcosis initiation, sevoflurane	90 (23.1%), n=390	1 (6.3%), n=16	0.14
Narcosis initiation, sufentanil	88 (22.6%), n=390	1 (6.3%), n=16	0.21
Narc. maintenance, midazolam	68 (16.3%), n=416	0 (0.0%), n=16	0.088
Narc. maintenance, propofol	104 (25.0%), n=416	2 (12.5%), n=16	0.38
Narc. maintenance, sevoflurane	409 (98.3%), n=416	16 (100.0%), n=16	1
Intracardiac device	23 (5.0%), n=463	3 (14.3%), n=21	0.096
Arterial hypertension	425 (87.4%), n=486	20 (95.2%), n=21	0.5
Diabetes mellitus	152 (31.3%), n=486	7 (35.0%), n=20	0.81
Hyperlipidemia	273 (56.2%), n=486	13 (61.9%), n=21	0.66
Smoking	200 (41.2%), n=486	7 (33.3%), n=21	0.51
NYHA classification	2.28 ± 0.802, n=429	2.42 ± 0.607, n=19	0.49
Stroke	37 (7.6%), n=485	2 (9.5%), n=21	0.67
Transient ischemic attack	15 (2.7%), n=485	0 (0.0%), n=21	1
Sleep apnea	31 (6.4%), n=482	2 (9.5%), n=21	0.64
COPD	55 (11.3%), n=485	4 (19.0%), n=21	0.29
Peripheral vascular disease	168 (21.9%), n=484	6 (30.0%), n=20	0.41
Heart rate pre-OP	68.9 ± 11.9, n=469	69.7 ± 12.8, n=20	0.77
Heart rate post-OP	74 ± 10.9, n=479	86.2 ± 15.4, n=21 ***	0.00018
Creatinine pre-OP (µmol/L)	1.01 ± 0.377, n=487	1.12 ± 0.387, n=21	0.056
Hemoglobin post-OP (g/dL)	10.3 ± 1.22, n=485	10.5 ± 1.2, n=21	0.36
Leucocytes post-OP (10^3/µL)	10.6 ± 3.37, n=485	11.3 ± 3.5, n=21	0.42
K^+ pre-OP (mmol/L)	4.14 ± 0.386, n=459	4.18 ± 0.463, n=21	0.58
K^+ post-OP (mmol/L)	4.22 ± 0.332, n=482	4.41 ± 0.327, n=21 *	0.013
Mg^{2+} pre OP (mmol/L)	0.957 ± 0.2, n=429	1.02 ± 0.264, n=18	0.44
Mg^{2+} post-OP (mmol/L)	0.841 ± 0.149, n=420	0.882 ± 0.132, n=17	0.14
Reduced eGFR post-OP	80 (16.7%), n=479	8 (38.1%), n=21 *	0.019
C-reactive protein post-OP (mg/L)	80.9 ± 43.4, n=482	66.5 ± 29.1, n=21	0.32
N stenosed vessels	2.53 ± 0.908, n=484	2.48 ± 0.981, n=21	0.79
RCA stenosis ≥70%	303 (63.3%), n=479	13 (65.0%), n=20	1
Previous myocardial infarction	0.443 ± 0.572, n=476	0.2 ± 0.41, n=20	0.055
Aortic root (mm)	33.2 ± 4.91, n=421	34.7 ± 3.64, n=18	0.16
LA diameter (mm)	41.4 ± 5.97, n=448	44.8 ± 6.24, n=18 **	0.008
IVSd (mm)	12.6 ± 2.44, n=464	13.8 ± 2.25, n=19 *	0.025
LVPWd (mm)	11.9 ± 2.37, n=448	13.2 ± 2.27, n=17 *	0.027
LVEDD (mm)	49 ± 7.36, n=464	49.2 ± 8.43, n=18	0.63
LVESD (mm)	33.2 ± 19.2, n=374	33.3 ± 8.87, n=16	0.7
LVEF (%)	52.2 ± 11.2, n=481	49.7 ± 11.6, n=19	0.3
LVH	361 (76.5%), n=472	18 (90.0%), n=20	0.27
Right ventricular diameter (mm)	36.2 ± 5.27, n=319	37.1 ± 4.95, n=10	0.7
intra-cardiac shunt	33 (7.9%), n=418	3 (14.3%), n=21	0.4

Pericardial effusion post-OP	182 (39.3%), n=463	10 (47.6%), n=21	0.5
Tricuspid valve insufficiency (I-III°)	0.695 ± 0.591, n=485	0.667 ± 0.483, n=21	0.97
Aortic valve stenosis (I-III°)	0.612 ± 1.12, n=484	1.45 ± 1.36, n=20 ***	0.00076
Aortic valve insufficiency (I-III°)	0.582 ± 0.795, n=486	0.714 ± 1.01, n=21	0.82
Mitral valve insufficiency (I-III°)	0.959 ± 0.699, n=486	1 ± 0.562, n=20	0.67
TAPSE (mm)	21.3 ± 6.13, n=380	21.1 ± 5.18, n=17	0.87
Diastolic dysfunction	0.962 ± 0.719, n=422	0.941 ± 0.659, n=17	0.97
E/A ratio	1.13 ± 0.47, n=340	1.24 ± 0.555, n=13	0.49
E/e' ratio	9.2 ± 3.72, n=310	12.1 ± 4.88, n=13 *	0.034
ACE inhibitor	244 (50.5%), n=483	4 (19.0%), n=21 **	0.0062
AT1 blocker	124 (25.7%), n=482	10 (47.6%), n=21 *	0.04
Metoprolol	80 (16.4%), n=487	5 (23.8%), n=21	0.37
Bisoprolol	190 (39.0%), n=487	7 (33.3%), n=21	0.66
Nebivolol	18 (3.7%), n=487	2 (9.5%), n=21	0.2
Other beta blocker	18 (3.7%), n=487	0 (0.0%), n=21	1
Calcium antagonist	124 (25.6%), n=484	9 (42.9%), n=21	0.12
Diuretic drug	161 (33.3%), n=484	12 (57.1%), n=21 *	0.033
Nitrate drug	57 (11.8%), n=484	2 (9.5%), n=21	1
Lipid lowering drug	374 (77.3%), n=484	14 (66.7%), n=21	0.29
ASS	344 (70.6%), n=487	17 (81.0%), n=21	0.46
Clopidogrel	40 (8.2%), n=487	2 (9.5%), n=21	0.69
Other aggregation inhibitor	24 (4.9%), n=487	0 (0.0%), n=21	0.62
Other anti-diabetic drug, incl. insulin	79 (16.0%), n=482	5 (23.8%), n=21	0.36
Metformin	70 (14.5%), n=482	4 (19.0%), n=21	0.53
Glucocorticoid	21 (4.4%), n=482	3 (14.3%), n=21	0.072
Mg^{2+} substitution	411 (84.4%), n=487	20 (95.2%), n=21	0.23
K^{+} substitution	446 (91.6%), n=487	21 (100.0%), n=21	0.4

For continuous parameters and ordinal parameters with more than two levels, means and standard deviations are given, for binary parameters, total counts and percentages are indicated; *p<0.05, **p<0.01, ***p<0.001 versus SR from Wilcoxon rank sum test for continuous variables and from Fisher exact test for categorical variables. ACE – angiotensin converting enzyme, ASS – acetylsalicylic acid, AT1 – angiotensin receptor 1, COPD – chronic obstructive pulmonary disease, eGFR – estimated glomerular filtration rate, IVSd – interventricular septum, LA – left atrium, LVEDD – left ventricular end-diastolic diameter, LVEF – left ventricular ejection fraction, LVESD – left ventricular end-systolic diameter, LVH – left ventricular hypertrophy, LVPWd – left posterior wall, NYHA – New York Heart Association, TAPSE – tricuspid annular plane systolic excursion

Supplementary Table S7. Comparisons of clinical parameters between during rehabilitation SR and AF groups.

	**SR during rehabilitation**, n=347	**AF during rehabilitation**, n=63 (15,4%)	*p*-value
Post-OP AF	96 (27.8%), n=345	44 (69.8%), n=63 ***	$5.4 \cdot 10^{-10}$
AF at discharge	2 (0.6%), n=331	16 (25.8%), n=62 ***	$2.9 \cdot 10^{-12}$
Age	65.7 ± 9.94, n=347	68.8 ± 8.82, n=63 *	0.047
Male gender	289 (83.3%), n=347	51 (81.0%), n=63	0.72
Height (m)	1.74 ± 0.0819, n=344	1.72 ± 0.089, n=63	0.15
Body mass index (kg/m^2)	28.2 ± 4.76, n=344	29.3 ± 5.16, n=62	0.11
Coronary artery bypass grafting	287 (82.9%), n=346	55 (87.3%), n=63	0.46
Aortic valve replacement	89 (25.7%), n=346	21 (33.3%), n=63	0.22
Intervention time (min)	282 ± 166, n=326	265 ± 61.9, n=60	0.37
Bypass time (min)	125 ± 40.9, n=304	124 ± 38.9, n=58	0.84
Heparin (IE) during intervention	29.8 ± 7.23, n=283	31.6 ± 10.1, n=49	0.6
Narcosis initiation, etomidate	9 (3.2%), n=277	1 (1.9%), n=53	1
Narcosis initiation, midazolam	196 (70.8%), n=277	40 (75.5%), n=53	0.62
Narcosis initiation, propofol	5 (1.8%), n=277	2 (3.8%), n=53	0.31
Narcosis initiation, sevoflurane	67 (24.2%), n=277	9 (17.0%), n=53	0.29
Narcosis initiation, sufentanil	67 (24.2%), n=277	9 (17.0%), n=53	0.29
Narc. maintenance, midazolam	47 (15.9%), n=295	6 (10.9%), n=55	0.42
Narc. maintenance, propofol	71 (24.1%), n=295	13 (23.6%), n=55	1
Narc. maintenance, sevoflurane	291 (98.6%), n=295	55 (100.0%), n=55	1
Intracardiac device	19 (5.9%), n=322	5 (8.2%), n=61	0.56
Arterial hypertension	306 (88.4%), n=346	56 (88.9%), n=63	1
Diabetes mellitus	114 (33.0%), n=345	21 (33.9%), n=62	0.88
Hyperlipidemia	209 (60.4%), n=346	33 (52.4%), n=63	0.27
Smoking	147 (42.7%), n=344	16 (25.4%), n=63 *	0.011
NYHA classification	2.34 ± 0.817, n=299	2.33 ± 0.632, n=58	0.66
Stroke	25 (7.2%), n=345	5 (7.9%), n=63	0.8
Transient ischemic attack	15 (3.8%), n=345	1 (1.6%), n=63	0.71
Sleep apnea	22 (6.5%), n=340	4 (6.3%), n=63	1
COPD	45 (13.0%), n=345	9 (14.3%), n=63	0.84
Peripheral vascular disease	135 (21.3%), n=344	16 (26.2%), n=61	0.4
Heart rate pre-OP	68.8 ± 12.3, n=330	69 ± 12.1, n=61	0.76
Heart rate post-OP	74.2 ± 11.1, n=331	78.2 ± 15.2, n=62 *	0.022
Creatinine pre-OP (µmol/L)	1.04 ± 0.526, n=347	1.07 ± 0.651, n=63	0.77
Hemoglobin post-OP (g/dL)	10.3 ± 1.24, n=347	10.3 ± 1.16, n=63	0.83
Leucocytes post-OP (10^3/µL)	10.7 ± 3.47, n=347	10.3 ± 3.15, n=63	0.25
K^+ pre-OP (mmol/L)	4.11 ± 0.407, n=327	4.2 ± 0.41, n=62	0.14
K^+ post-OP (mmol/L)	4.22 ± 0.332, n=341	4.28 ± 0.319, n=61	0.37
Mg^{2+} prä OP (mmol/L)	0.968 ± 0.198, n=304	0.972 ± 0.252, n=58	0.49
Mg^{2+} post-OP (mmol/L)	0.844 ± 0.144, n=299	0.861 ± 0.174, n=53	0.64
Reduced eGFR post-OP	68 (19.7%), n=345	12 (19.0%), n=63	1
C-reactive protein post-OP (mg/L)	81.6 ± 43.4, n=344	76.6 ± 37.1, n=63	0.57
N stenosed vessels	2.55 ± 0.868, n=345	2.52 ± 0.913, n=63	0.99
RCA stenosis ≥70%	213 (62.8%), n=339	43 (68.3%), n=63	0.48
Previous myocardial infarction	0.457 ± 0.572, n=335	0.367 ± 0.486, n=60	0.32
Aortic root (mm)	33.2 ± 4.94, n=289	33.9 ± 4.55, n=56	0.25
LA diameter (mm)	41.2 ± 5.89, n=317	43.7 ± 6.37, n=57 **	0.0034
IVSd (mm)	12.5 ± 2.36, n=327	13.2 ± 2.46, n=58	0.06
LVPWd (mm)	11.9 ± 2.42, n=314	12.3 ± 2.29, n=55	0.15
LVEDD (mm)	48.8 ± 7.32, n=327	50 ± 7.38, n=60	0.19
LVESD (mm)	33.6 ± 22.7, n=256	32.3 ± 7.63, n=48	0.98
LVEF (%)	51.9 ± 11.2, n=342	52.4 ± 11.9, n=61	0.66
LVH	256 (76.2%), n=336	50 (82.0%), n=61	0.41

Right ventricular diameter (mm)	36.1 ± 5.15, n=214	36.8 ± 5.57, n=39	0.35
intra-cardiac shunt	26 (8.9%), n=291	5 (8.8%), n=57	1
Pericardial effusion post-OP	118 (36.5%), n=323	33 (54.1%), n=61 *	0.015
Tricuspid valve insufficiency (I-III°)	0.659 ± 0.584, n=346	0.726 ± 0.548, n=62	0.33
Aortic valve stenosis (I-III°)	0.564 ± 1.11, n=346	1.13 ± 1.31, n=61 ***	$3.9 \cdot 10^{-5}$
Aortic valve insufficiency (I-III°)	0.564 ± 0.767, n=346	0.613 ± 0.732, n=62	0.45
Mitral valve insufficiency (I-III°)	0.962 ± 0.707, n=346	1.07 ± 0.704, n=61	0.24
TAPSE (mm)	20.8 ± 5.82, n=266	22.5 ± 6.55, n=49	0.13
Diastolic dysfunction	0.964 ± 0.707, n=302	0.963 ± 0.751, n=54	0.97
E/A ratio	1.09 ± 0.465, n=231	1.2 ± 0.518, n=45	0.27
E/e ratio	9.21 ± 3.63, n=219	9.74 ± 4.12, n=32	0.38
ACE inhibitor	179 (51.6%), n=344	26 (41.9%), n=62	0.17
AT1 blocker	92 (26.8%), n=343	22 (35.5%), n=62	0.17
Metoprolol	55 (15.9%), n=347	12 (19.0%), n=63	0.58
Bisoprolol	133 (38.3%), n=347	28 (44.4%), n=63	0.4
Nebivolol	15 (4.3%), n=347	3 (4.8%), n=63	0.75
Other beta blocker	14 (4.0%), n=347	1 (1.6%), n=63	0.48
Calcium antagonist	92 (26.7%), n=345	18 (29.0%), n=62	0.76
Diuretic drug	120 (34.8%), n=345	26 (41.9%), n=62	0.31
Nitrate drug	38 (11.0%), n=345	9 (14.5%), n=62	0.4
Lipid lowering drug	276 (80.0%), n=345	46 (74.2%), n=62	0.31
ASS	247 (71.2%), n=347	44 (69.8%), n=63	0.88
Clopidogrel	28 (8.1%), n=347	6 (9.5%), n=63	0.63
Other aggregation inhibitor	20 (5.8%), n=347	0 (0.0%), n=63	0.054
Other anti-diabetic drug, incl. insulin	56 (15.7%), n=344	13 (21.0%), n=62	0.35
Metformin	50 (14.5%), n=344	12 (19.4%), n=62	0.34
Glucocorticoid	14 (4.1%), n=344	6 (9.7%), n=62	0.1
Mg^{2+} substitution	286 (82.4%), n=347	57 (90.5%), n=63	0.14
K^{+} substitution	314 (90.5%), n=347	61 (96.8%), n=63	0.14

For continuous parameters and ordinal parameters with more than two levels, means and standard deviations are given, for binary parameters, total counts and percentages are indicated; *$p<0.05$, **$p<0.01$, ***$p<0.001$ versus SR from Wilcoxon rank sum test for continuous variables and from Fisher exact test for categorical variables. ACE – angiotensin converting enzyme, ASS – acetylsalicylic acid, AT1 – angiotensin receptor 1, COPD – chronic obstructive pulmonary disease, eGFR – estimated glomerular filtration rate, IVSd – interventricular septum, LA – left atrium, LVEDD – left ventricular end-diastolic diameter, LVEF – left ventricular ejection fraction, LVESD – left ventricular end-systolic diameter, LVH – left ventricular hypertrophy, LVPWd – left posterior wall, NYHA – New York Heart Association, TAPSE – tricuspid annular plane systolic excursion

Supplementary Table S8. Comparisons of clinical parameters between patients with post-OP AF, AF at discharge or AF during rehabilitation.

	**Post-OP AF,** n=173	**AF at discharge,** n=21	**p** vs. poAF	***AF during rehabilitation,*** *n=63*	***p*** *vs. poAF*	***p*** *vs. disch.*
Age (y)	69.3 ± 9.0, n=173	71.4 ± 7.6, n=21	0.35	68.8 ± 8.8, n=63	0.56	0.23
Male gender	138 (79.8%), n=173	17 (81.0%), n=21	1	51 (79.8%), n=63	1	1
Height (m)	1.74 ± 0.08, n=173	1.73 ± 0.09, n=21	0.8	1.72 ± 0.09, n=63	0.43	0.88
Body mass index (kg/m^2)	28.2 ± 4.5, n=173	29.8 ± 5.0, n=21	0.13	29.3 ± 5.2, n=62	0.19	0.56
Coronary artery bypass grafting	144 (83.2%), n=173	17 (81.0%), n=21	0.76	55 (83.2%), n=63	0.55	0.48
Aortic valve replacement	49 (28.3%), n=173	11 (52.4%), n=21 *	0.043	21 (28.3%), n=63	0.52	0.13
Intervention time (min)	290 ± 231, n=162	256 ± 73, n=20	0.31	265 ± 62, n=60	0.72	0.39
Bypass time (min)	128 ± 55, n=151	112 ± 35, n=17	0.26	124 ± 39, n=58	0.86	0.31
Heparin (IE) during intervention	30.1 ± 7.2, n=133	29.0 ± 4.8, n=17	0.67	31.6 ± 10.1, n=49	0.68	0.51
Narcosis initiation, etomidate	4 (2.9%), n=139	0 (0.0%), n=16	1	1 (2.9%), n=53	1	1
Narcosis initiation, midazolam	96 (69.1%), n=139	14 (87.5%), n=16	0.15	40 (69.1%), n=53	0.48	0.49
Narcosis initiation, propofol	4 (2.9%), n=139	0 (0.0%), n=16	1	2 (2.9%), n=53	0.67	1
Narcosis initiation, sevoflurane	32 (23.0%), n=139	1 (6.3%), n=16	0.2	9 (23.0%), n=53	0.43	0.43
Narcosis initiation, sufentanil	32 (23.0%), n=139	1 (6.3%), n=16	0.2	9 (23.0%), n=53	0.43	0.43
Narc. maintenance, midazolam	19 (13.3%), n=143	0 (0.0%), n=16	0.22	6 (13.3%), n=55	0.81	0.33
Narc. maintenance, propofol	40 (28.0%), n=143	2 (12.5%), n=16	0.24	13 (28.0%), n=55	0.59	0.49
Narc. maintenance, sevoflurane	141 (98.6%), n=143	16 (100.0%), n=16	1	55 (98.6%), n=55	1	1
Intracardiac device	14 (8.6%), n=162	3 (14.3%), n=21	0.42	5 (8.6%), n=61	1	0.42
Arterial hypertension	154 (89.0%), n=173	20 (95.2%), n=21	0.7	56 (89.0%), n=63	1	0.67
Diabetes mellitus	60 (35.1%), n=171	7 (35.0%), n=20	1	21 (35.1%), n=62	1	1
Hyperlipidemia	94 (54.3%), n=173	13 (61.9%), n=21	0.64	33 (54.3%), n=63	0.88	0.61
Smoking	55 (32.0%), n=172	7 (33.3%), n=21	1	16 (32.0%), n=63	0.42	0.57
NYHA classification	2.43 ± 0.67, n=156	2.42 ± 0.61, n=19	0.92	2.33 ± 0.63, n=58	0.29	0.59
Stroke	15 (8.8%), n=171	2 (9.5%), n=21	1	5 (8.8%), n=63	1	1
Transient ischemic attack	8 (3.5%), n=171	0 (0.0%), n=21	1	1 (3.5%), n=63	0.68	1
Sleep apnea	12 (7.1%), n=168	2 (9.5%), n=21	0.66	4 (7.1%), n=63	1	0.64
COPD	24 (14.0%), n=172	4 (19.0%), n=21	0.52	9 (14.0%), n=63	1	0.73
Peripheral vascular disease	40 (23.5%), n=170	6 (30.0%), n=20	0.58	16 (23.5%), n=61	0.73	0.78
Heart rate pre-OP	69.4 ± 11.6, n=162	69.7 ± 12.8, n=20	0.85	69.0 ± 12.1, n=61	1	0.86
Heart rate post-OP	74.8 ± 13, n=157	86.2 ± 15.4, n=21 **	0.0013	78.2 ± 15.2, n=62	0.09	0.051
Creatinine pre-OP (µmol/L)	1.07 ± 0.57, n=173	1.12 ± 0.39, n=21	0.15	1.07 ± 0.65, n=63	0.9	0.14
Hemoglobin post-OP (g/dL)	10.3 ± 1.2, n=173	10.5 ± 1.2, n=21	0.42	10.3 ± 1.2, n=63	0.89	0.38
Leucocytes post-OP (10^3/µL)	10.6 ± 3.2, n=173	11.3 ± 3.5, n=21	0.48	10.3 ± 3.2, n=63	0.33	0.26
K^+ pre-OP (mmol/L)	4.14 ± 0.41, n=167	4.18 ± 0.46, n=21	0.61	4.2 ± 0.4, n=62	0.37	0.96
K^+ post-OP (mmol/L)	4.29 ± 0.32, n=168	4.41 ± 0.33, n=21	0.14	4.28 ± 0.32, n=61	0.66	0.11
Mg^{2+} prä OP (mmol/L)	0.97 ± 0.21, n=153	1.02 ± 0.26, n=18	0.57	0.97 ± 0.25, n=58	0.65	0.59
Mg^{2+} post-OP (mmol/L)	0.89 ± 0.21, n=155	0.88 ± 0.13, n=17	0.72	0.86 ± 0.17, n=53	0.34	0.32
Reduced eGFR post-OP	49 (28.7%), n=171	8 (38.1%), n=21	0.45	12 (28.7%), n=63	0.18	0.086
C-reactive protein post-OP (mg/L)	77.4 ± 40.1, n=173	66.5 ± 29.1, n=21	0.47	76.6 ± 37.1, n=63	0.97	0.55
N stenosed vessels	2.5 ± 0.91, n=172	2.48 ± 0.98, n=21	0.97	2.52 ± 0.91, n=63	0.72	0.8
RCA stenosis ≥70%	103 (61.7%), n=167	13 (65.0%), n=20	1	43 (61.7%), n=63	0.44	0.79
Previous myocardial infarction	0.42 ± 0.53, n=163	0.20 ± 0.41, n=20	0.081	0.37 ± 0.49, n=60	0.6	0.17
Aortic root (mm)	33.5 ± 5.1, n=149	34.7 ± 3.6, n=18	0.24	33.9 ± 4.6, n=56	0.44	0.51
LA diameter (mm)	42.8 ± 6.5, n=164	44.8 ± 6.2, n=18	0.098	43.7 ± 6.4, n=57	0.27	0.33
IVSd (mm)	12.6 ± 2.5, n=161	13.8 ± 2.3, n=19 *	0.039	13.2 ± 2.5, n=58	0.13	0.31
LVPWd (mm)	11.9 ± 2.3, n=156	13.2 ± 2.3, n=17 *	0.022	12.3 ± 2.3, n=55	0.16	0.15
LVEDD (mm)	49.5 ± 8.5, n=166	49.2 ± 8.4, n=18	0.86	50 ± 7.4, n=60	0.53	0.91
LVESD (mm)	32.5 ± 7.9, n=140	33.3 ± 8.9, n=16	0.73	32.3 ± 7.6, n=48	0.94	0.68
LVEF (%)	50.2 ± 12.3, n=170	49.7 ± 11.6, n=19	0.79	52.4 ± 11.9, n=61	0.19	0.29
LVH	130 (78.3%), n=166	18 (90.0%), n=20	0.38	50 (78.3%), n=61	0.59	0.5

Right ventricular diameter (mm)	36.4 ± 5.5, n=108	37.1 ± 5.0, n=10	0.87	36.8 ± 5.6, n=39	0.66	0.96
Intra-cardiac shunt	11 (7.2%), n=152	3 (14.3%), n=21	0.38	5 (7.2%), n=57	0.77	0.67
Pericardial effusion post-OP	71 (44.1%), n=161	10 (47.6%), n=21	0.82	33 (44.1%), n=61	0.23	0.62
Tricuspid valve insufficiency (I-III°)	0.79 ± 0.59, n=172	0.67 ± 0.48, n=21	0.41	0.73 ± 0.55, n=62	0.5	0.73
Aortic valve stenosis (I-III°)	0.79 ± 1.25, n=172	1.45 ± 1.36, n=20 *	0.019	1.13 ± 1.31, n=61 *	0.032	0.37
Aortic valve insufficiency (I-III°)	0.61 ± 0.83, n=172	0.71 ± 1.01, n=21	0.88	0.61 ± 0.73, n=62	0.65	0.96
Mitral valve insufficiency (I-III°)	1.06 ± 0.72, n=171	1.00 ± 0.56, n=20	0.83	1.07 ± 0.70, n=61	0.85	0.74
TAPSE (mm)	21.9 ± 6.3, n=134	21.1 ± 5.2, n=17	0.74	22.5 ± 6.6, n=49	0.56	0.5
Diastolic dysfunction	0.94 ± 0.70, n=147	0.94 ± 0.66, n=17	0.96	0.96 ± 0.75, n=54	0.9	0.99
E/A ratio	1.15 ± 0.47, n=114	1.24 ± 0.56, n=13	0.56	1.2 ± 0.52, n=45	0.61	0.81
E/e ratio	9.7 ± 3.7, n=89	12.1 ± 4.9, n=13	0.11	9.7 ± 4.1, n=32	0.89	0.18
ACE inhibitor	82 (46.8%), n=172	4 (19.0%), n=21 *	0.019	26 (46.8%), n=62	0.55	0.07
AT1 blocker	42 (24.6%), n=171	10 (47.6%), n=21 *	0.036	22 (24.6%), n=62	0.13	0.44
Metoprolol	32 (18.5%), n=173	5 (23.8%), n=21	0.56	12 (18.5%), n=63	1	0.75
Bisoprolol	68 (39.3%), n=173	7 (33.3%), n=21	0.64	28 (39.3%), n=63	0.55	0.45
Nebivolol	10 (5.8%), n=173	2 (9.5%), n=21	0.62	3 (5.8%), n=63	1	0.59
Other beta blocker	7 (4.0%), n=173	0 (0.0%), n=21	1	1 (4.0%), n=63	0.69	1
Calcium antagonist	56 (32.6%), n=172	9 (42.9%), n=21	0.34	18 (32.6%), n=62	0.64	0.29
Diuretic drug	75 (43.6%), n=172	12 (57.1%), n=21	0.25	26 (43.6%), n=62	0.88	0.31
Nitrate drug	19 (11.0%), n=172	2 (9.5%), n=21	1	9 (11.0%), n=62	0.5	0.72
Lipid lowering drug	130 (75.6%), n=172	14 (66.7%), n=21	0.43	46 (75.6%), n=62	0.86	0.58
ASS	124 (71.7%), n=173	17 (81.0%), n=21	0.45	44 (71.7%), n=63	0.87	0.41
Clopidogrel	11 (6.4%), n=173	2 (9.5%), n=21	0.64	6 (6.4%), n=63	0.4	1
Other aggregation inhibitor	7 (4.0%), n=173	0 (0.0%), n=21	1	0 (4.0%), n=63	0.19	1
Other anti-diabetic drug, incl. insulin	37 (20.6%), n=171	5 (23.8%), n=21	0.78	13 (20.6%), n=62	1	0.77
Metformin	30 (17.5%), n=171	4 (19.0%), n=21	0.77	12 (17.5%), n=62	0.85	1
Glucocorticoid	14 (8.2%), n=171	3 (14.3%), n=21	0.41	6 (8.2%), n=62	0.79	0.69
Mg^{2+} substitution	147 (85.0%), n=173	20 (95.2%), n=21	0.32	57 (85.0%), n=63	0.39	0.67
K^+ substitution	161 (93.1%), n=173	21 (100.0%), n=21	0.37	61 (93.1%), n=63	0.36	1

For continuous parameters and ordinal parameters with more than two levels, means and standard deviations are given, for binary parameters, total counts and percentages are indicated. Numbers *n* indicate patients for whom parameters were available; p-values are indicated for comparisons with post-OP AF and with AF at discharge (disch.), **p*<0.05, ***p*<0.01 versus post-OP AF from Wilcoxon rank sum test for continuous variables and of Fisher exact test for categorical variables. ACE – angiotensin converting enzyme, ASS – acetylsalicylic acid, AT1 – angiotensin receptor 1, COPD – chronic obstructive pulmonary disease, eGFR – estimated glomerular filtration rate, IVSd – interventricular septum, LA – left atrium, LVEDD – left ventricular end-diastolic diameter, LVEF – left ventricular ejection fraction, LVESD – left ventricular end-systolic diameter, LVH – left ventricular hypertrophy, LVPWd – left posterior wall, NYHA – New York Heart Association, TAPSE – tricuspid annular plane systolic excursion.

Supplementary Table S9. Model coefficients and odds ratios for predicting postoperative AF from clinical parameters.

	Postoperative AF vs. SR			
	Coefficient (95% CI)	Odds ratio (95% CI)	Variable increment	*p*-value
Age (y)	0.321 (0.103, 0.539)	1.378 (1.108, 1.715)	10 years	0.0040
LA diameter (mm)	0.227 (0.066, 0.388)	1.254 (1.068, 1.473)	5 mm	0.0058
Glucocorticoid intake	1.177 (0.307, 2.046)	3.243 (1.359, 7.737)		0.0080
Mg^{2+} post-OP (mmol/L)	0.160 (0.038, 0.282)	1.173 (1.038, 1.326)	0.1 mmol/L	0.0104
Reduced post-OP. eGFR	0.591 (0.106, 1.075)	1.805 (1.112, 2.931)		0.0169
Aortic stenosis (I-III°)	0.176 (0.011, 0.341)	1.192 (1.011, 1.407)	degree	0.0370
Nicotine	-0.440 (-0.869, -0.010)	0.644 (0.419, 0.990)		0.0447
Tricuspid valve insufficiency (I-III°)	0.325 (0.002, 0.648)	1.385 (1.002, 1.913)	degree	0.0484
Intercept	-6.506 (-8.757, -4.254)			$1.48 \cdot 10^{-8}$

LA, left atrial; eGFR, estimated glomerular filtration rate. Model parameters were ordered according to *p*-values, numeric parameters were scaled to representative variable increments as indicated in the fourth column.

Supplementary Table S10. Model coefficients and odds ratios for predicting AF at discharge from clinical parameters.

	AF at discharge vs. SR			
	Coefficient (95% CI)	Odds ratio (95% CI)	Variable increment	p-value
Heart rate (1/min)	0.933 (0.533, 1.334)	2.543 (1.703, 3.796)	10/min	$4.97 \cdot 10^{-6}$
Age (y)	1.125 (0.462, 1.789)	3.081 (1.587, 5.985)	10 years	$8.91 \cdot 10^{-4}$
Aortic valve replacement	1.588 (0.524, 2.652)	4.893 (1.688, 14.182)		0.00345
BMI	0.693 (0.176, 1.209)	1.999 (1.192, 3.352)	5 kg/m²	0.00862
AT1 blocker intake	1.236 (0.191, 2.281)	3.442 (1.211, 9.785)		0.0204
Previous myocardial infarctions	-1.305 (-2.545, -0.066)	0.271 (0.078, 0.936)	1	0.0390
Intercept	-23.1 (-31.1, -15.062)			$1.70 \cdot 10^{-8}$

BMI, body mass index. Model parameters were ordered according to p-values, numeric parameters were scaled to representative variable increments as indicated in the fourth column.

Supplementary Table S11. Model coefficients and odds ratios for predicting AF during rehab from clinical parameters.

	**AF during rehab vs. SR**			
	Coefficient (95% CI)	Odds ratio (95% CI)	Variable increment	p-value
Aortic valve stenosis (I-III°)	0.395 (0.175, 0.614)	1.484 (1.192, 1.848)	degree	$4.22 \cdot 10^{-4}$
LA diameter (mm)	0.347 (0.117, 0.578)	1.415 (1.125, 1.782)	5 mm	0.0031
Nicotine	-0.952 (-1.597, -0.307)	0.386 (0.203, 0.736)		0.0038
Heart rate (1/min)	0.271 (0.432, 0.499)	1.312 (1.044, 1.647)	10/min	0.0197
PE post-OP	0.666 (0.093, 1.239)	1.947 (1.098, 3.452)		0.0226
Intercept	-7.013 (-9.870, -4.157)			$1.49 \cdot 10^{-6}$

LA, left atrial; PE, pericardial effusion. Model parameters were ordered according to p-values, numeric parameters were scaled to representative variable increments as indicated in the fourth column.

Supplementary Table S12. Predicting AF at discharge from clinical parameters including post-OP AF.

	**AF at discharge vs. SR**			
	Coefficient (95% CI)	Odds ratio (95% CI)	Variable increment	p-value
Heart rate (1/min)	0.922 (0.426, 1.418)	2.514 (1.530, 4.131)	10/min	$2.73 \cdot 10^{-4}$
AT1 blocker intake	1.585 (0.427, 2.743)	4.879 (1.532, 15.535)		$7.32 \cdot 10^{-3}$
Aortic stenosis (I-III°)	0.521 (0.097, 0.946)	1.684 (1.102, 2.574)	degree	0.0160
E/e' ratio	0.156 (0.027, 0.286)	1.169 (1.027, 1.332)	1	0.0183
Previous myocardial infarctions	-1.645 (-3.079, -0.210)	0.193 (0.046, 0.810)	1	0.0246
Post-OP AF	101 ($-7.02 \cdot 10^{-6}$, $7.02 \cdot 10^{6}$)	$6.31 \cdot 10^{-43}$ (0, Inf)		n. a.
Intercept	-112 ($-7.02 \cdot 10^{-6}$, $7.02 \cdot 10^{6}$)			n. a.

E/e’ ratio, ratio of early diastolic mitral filling velocity to early diastolic mitral annulus velocity; AT1 blocker, angiotensin 1 receptor blocker. Model parameters were ordered according to p-values, numeric parameters were scaled to representative variable increments as indicated in the fourth column. For the parameter ‘post-OP AF’ and the intercept, no valid confidence interval or p-value could be estimated because all subjects with AF at discharge had post-OP AF.

Supplementary Table S13. Predicting AF during rehab from clinical parameters including post-OP AF and AF at discharge.

	**AF during rehab vs. SR**			
	Coefficient (95% CI)	Odds ratio (95% CI)	Variable increment	p-value
AF at discharge	3.362 (1.777, 4.948)	28.85 (5.91, 140.82)		$3.24 \cdot 10^{-5}$
Post-OP AF	1.167 (0.521, 1.813)	3.212 (1.683, 6.129)		$4.01 \cdot 10^{-4}$
Aortic valve stenosis (I-III°)	0.290 (0.046, 0.534)	1.336 (1.047, 1.705)	degree	0.0200
Nicotine	-0.822 (-1.533, -0.110)	0.440 (0.216, 0.896)		0.0236
PE post-OP	0.665 (0.033, 1.296)	1.944 (1.034, 3.656)		0.0391
Intercept	-2.745 (-3.364, -2.127)			$3.2 \cdot 10^{-18}$

PE, pericardial effusion. Model parameters were ordered according to p-values, numeric parameters were scaled to representative variable increments as indicated in the fourth column.

Supplementary Figures

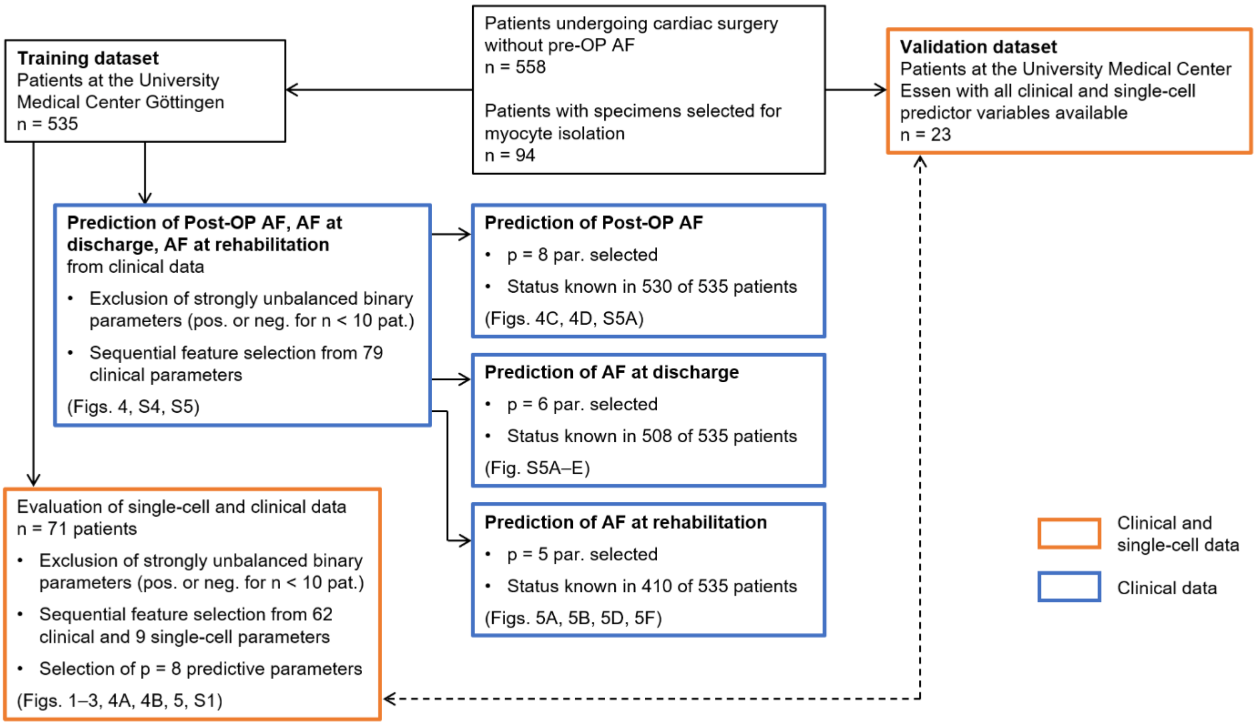

Figure S1. Visualisation of the study dataset. The study comprises a training dataset of patients undergoing cardiac surgery (n=535 patients). In a subset of n=71 patients, clinical and single-cell data were available. From this subset, a model for predicting post-OP AF was developed and tested in a validation dataset from another medical centre containing values from n=23 patients with all required clinical and single-cell parameters (orange boxes). Based on the larger datasets of clinical parameters, models for prediction of post-OP AF, AF at discharge, and AF at rehabilitation were developed (blue boxes). Due to the exclusion of strongly unbalanced binary variables, fewer clinical predictors (n=62) were included in the subset of clinical and single-cell parameters than in the larger set of clinical parameters (n=79). Sequential feature selection was applied to determine most important parameters in models for prediction of post-OP AF, AF at discharge or AF at rehabilitation. par. = parameters, pos. = positive, neg. = negative

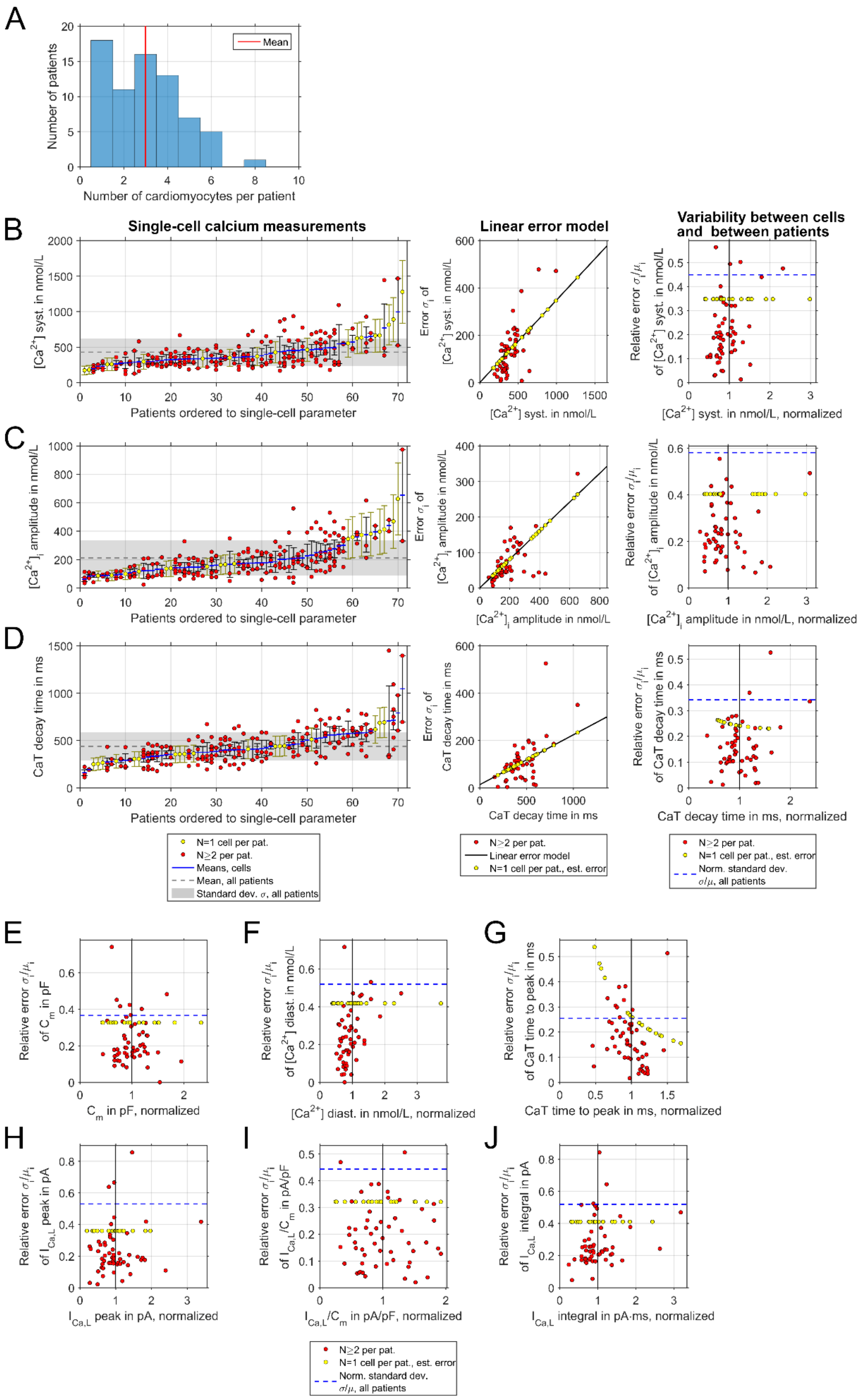

Figure S2. Variability of single-cell calcium measurements. Evaluating single-cell calcium measurements showed that the variability between means of single-cell measurements in patients was, in most cases, larger than the dispersion of the means of cardiomyocyte measurements in single subjects. Comparisons are indicated for three parameters with

significant differences between post-OP AF and SR groups ($[Ca^{2+}]_{syst.}$, $[Ca^{2+}]_i$ amplitude, and the CaT decay time). (**A**) Histogram of cells measured per patient (median and mean equal 3). (**B**) ***left*:** single-cell systolic calcium concentration measurements $[Ca^{2+}]_{syst.}$ obtained from cardiomyocytes of patients (n=1…8 cell per patient; black error bars, SEM in patients with n≥2 cells; yellow error bars, estimated errors in patients with n=1 cell). ***Centre*:** A linear error model was fitted to standard error (SE) values of patients with n≥3 cells (black line). Red circles indicate SE values of patients with n≥2 cells against $[Ca^{2+}]_{syst.}$ measurements. Yellow circles indicate estimated errors of patients with n=1 cell obtained from the linear error model. ***Right*:** SE of all patients normalised by the overall mean, equal to the coefficient of variation (blue dashed line), indicated together with relative errors of single-cell means of single patients (SEM values divided by mean values). In case of patients with n=1 cell, errors estimated from the linear error model are indicated. (**C**) Measurements, estimated errors and linear error model for $[Ca^{2+}]$ amplitude as in (B). (**D**) Measurements, estimated errors and linear error model for CaT decay time as in (B). (**E–J**) SE of all patients normalized by the overall mean (blue dashed line), and relative errors of single-cell means of single patients (red circles, SEM values divided by mean values; yellow circles, errors estimated from linear error model for patients with n=1 cell) for (**E**) C_m, (**F**) $[Ca^{2+}]_{diast.}$, (**G**) CaT time to peak, (**H**) $I_{Ca,L}$ peak, (**I**) $I_{Ca,L}/C_m$, and (**J**) the integral of $I_{Ca,L}$.

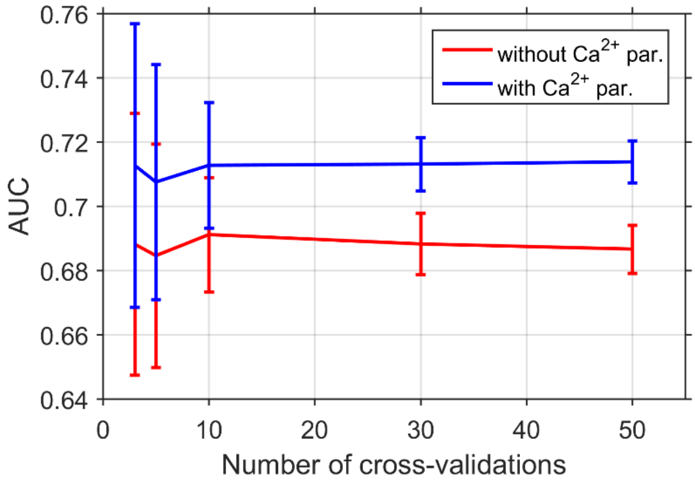

Figure S3. AUC value increase for including the most predictive single-cell parameter. Differences in the AUC values of ROC curves for predicting post-OP AF from a combination of clinical and single-cell parameters were assessed for different values of k for k-fold cross-validation. To test the difference, k-fold cross-validation was performed 100 times with random assignments to training and test datasets. Error bars indicate means and standard deviations of AUC values. In case of 3-, 10-, 30- and 50-fold cross-validation, the mean AUC value without the additional Ca^{2+} parameter ($[Ca^{2+}]_{syst}$) was 0.69, and 0.71 when including the Ca^{2+} parameter. In case of 5-fold cross-validation the mean AUC value without the additional Ca^{2+} parameter was 0.68, and 0.71 when including the Ca^{2+} parameter. In all cases, including the most predictive single-cell parameter resulted in a significant AUC increase ($p<10^{-4}$ from Wilcoxon rank sum test).

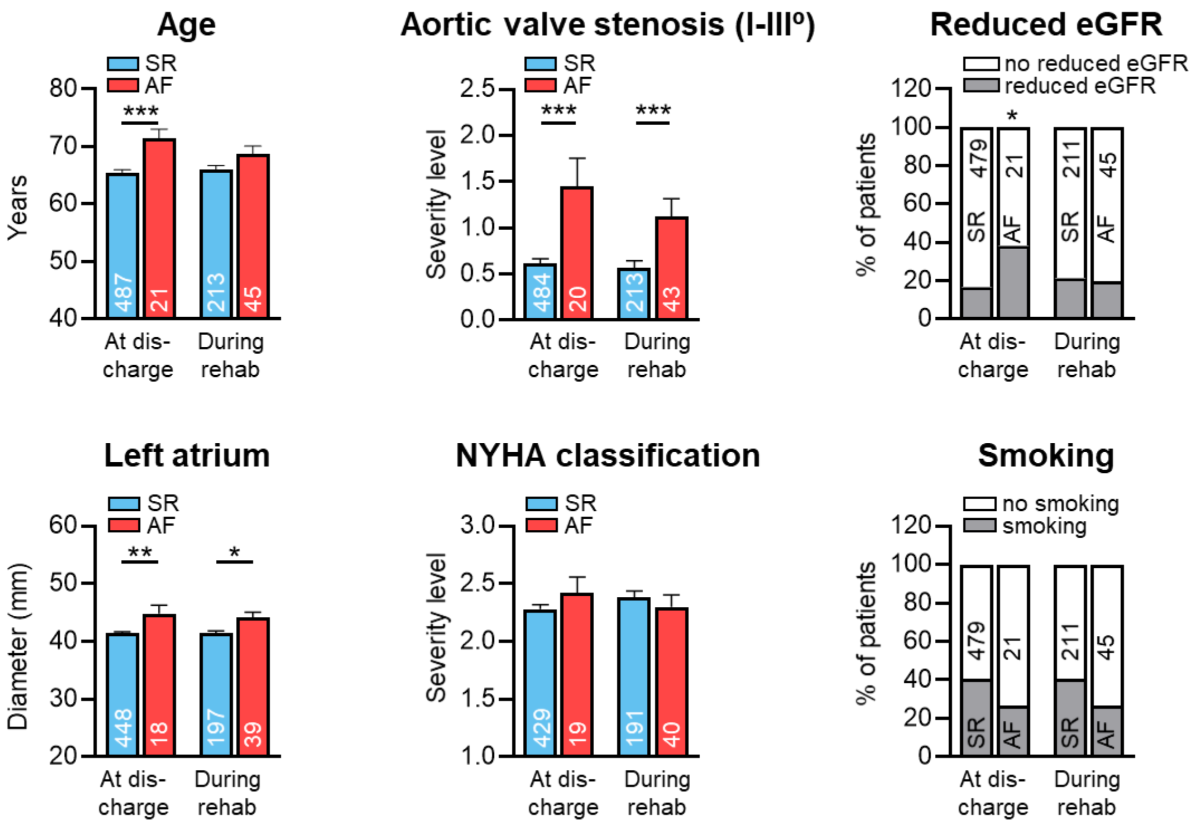

Supplementary Figure S4. **Atrial fibrillation (AF) at discharge and during rehabilitation periods following cardiac surgery**. Selected clinical characteristics of patients with AF at discharge and during rehabilitation periods following cardiac surgery compared to patients with sinus rhythm (SR). Data are presented as mean±standard deviation or percentages of patients. *p<0.05, **p<0.01, ***p<0.001 vs SR. Comparison using Wilcoxon rank sum test (see **Supplementary Tables S5 and S6** for details). eGFR, estimated glomerular filtration rate; NYHA, New York Heart Association.

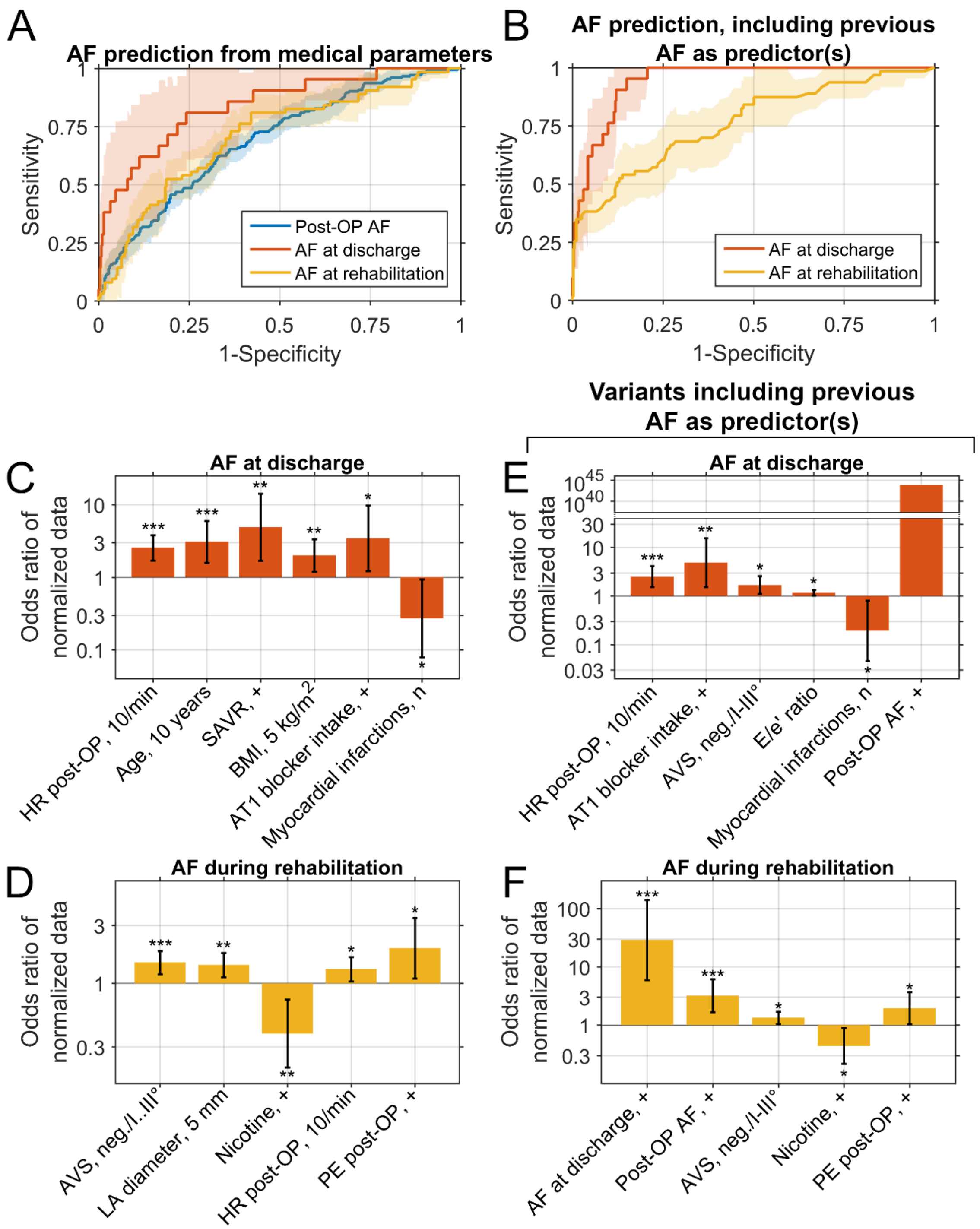

Supplementary Figure S5. **Prediction of postoperative AF based on clinical parameters.** Logistic regression models were calibrated using medical history and clinical parameters. Predictive parameters were selected based on sequential feature selection. (**A**) ROC curves for predicting post-OP AF (AUC=0.69; 95% CI, [0.66, 0.72]), AF at discharge (AUC=0.84; 95% CI, [0.73, 0.95]) or during rehabilitation treatment (AUC=0.71; 95% CI, [0.65, 0.76]; areas: 95% confidence intervals estimated from 10-fold cross-validation). (**B**) ROC curves for predictions including AF at previous times, i. e., prediction of AF at discharge including post-OP AF as predictor (AUC=0.94; 95% CI, [0.92, 0.97]), and prediction of AF during rehabilitation treatment (AUC=0.76; 95% CI, [0.70, 0.83]) including post-OP AF and AF at discharge as predictors as in (A). (**C**–**F**) Bars indicate odds ratios, i. e., ratios between the chance of developing AF to the chance of not developing AF, for (**C**) AF at time of discharge, (**D**) during a rehabilitation treatment, (**E**) AF at discharge including post-OP AF during in-hospital post-OP period as predictor, and (**F**) AF during rehabilitation treatment including post-OP AF during in-hospital

post-OP period and AF at discharge as predictors (error bars: 95% confidence intervals; *$p<0.05$, **$p<0.01$ ***$p<0.001$, see **Supplementary Tables S9-S12** for details). In (**E**), the value for the parameter ‘post-OP AF’ was not identifiable because all patients with AF at discharge had post-OP AF. In bar graphs, parameters were sorted from small (left) to larger *p*-values (right). Odds ratios reflect the effects of binary variable changes, indicated by a ‘+’ or continuous variable changes by indicated unit intervals or numbers n. AVS, aortic valve stenosis; HR, heart rate; LA, left atrium; PE, pericardial effusion; SAVR, surgical aortic valve replacement; TI, tricuspid valve insufficiency.

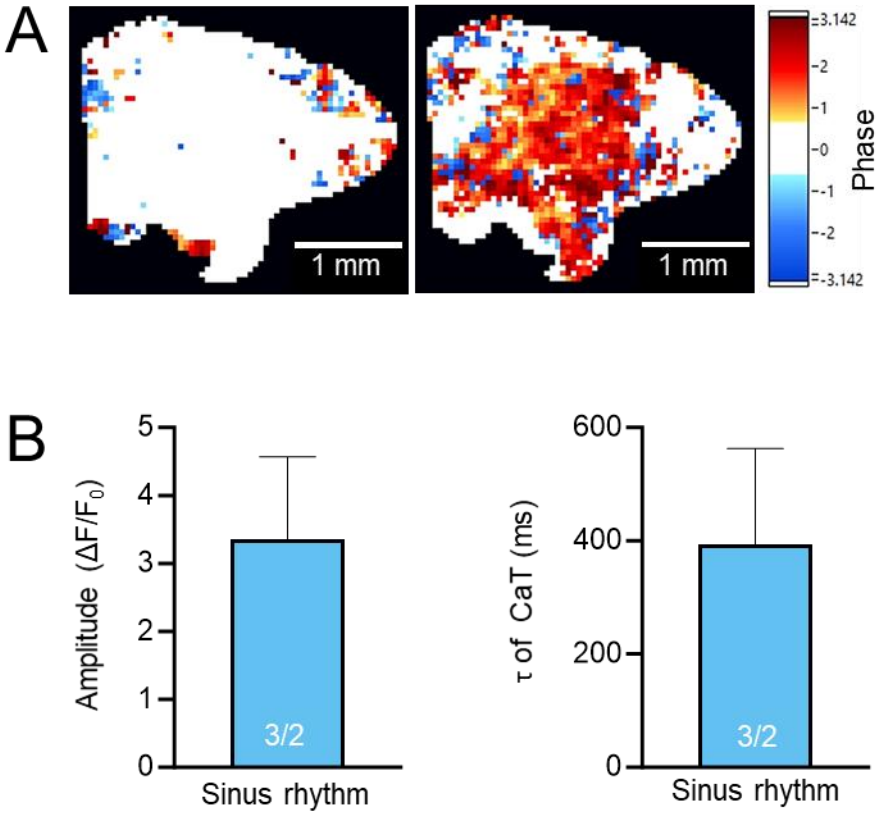

Supplementary Figure S6. Measurement of cytosolic calcium in trabeculae carneae from human atrial appendages. (**A**) Phase maps of a trabecula during diastole (*left*) and systole (*right*) calculated using Hilbert-transform. (**B**) Mean±standard deviation of calculated amplitudes (*left*) and time constant of decay (τ, *right*) of calcium transient (CaT). Numbers within columns indicate trabeculae/patients.

Supplementary Video

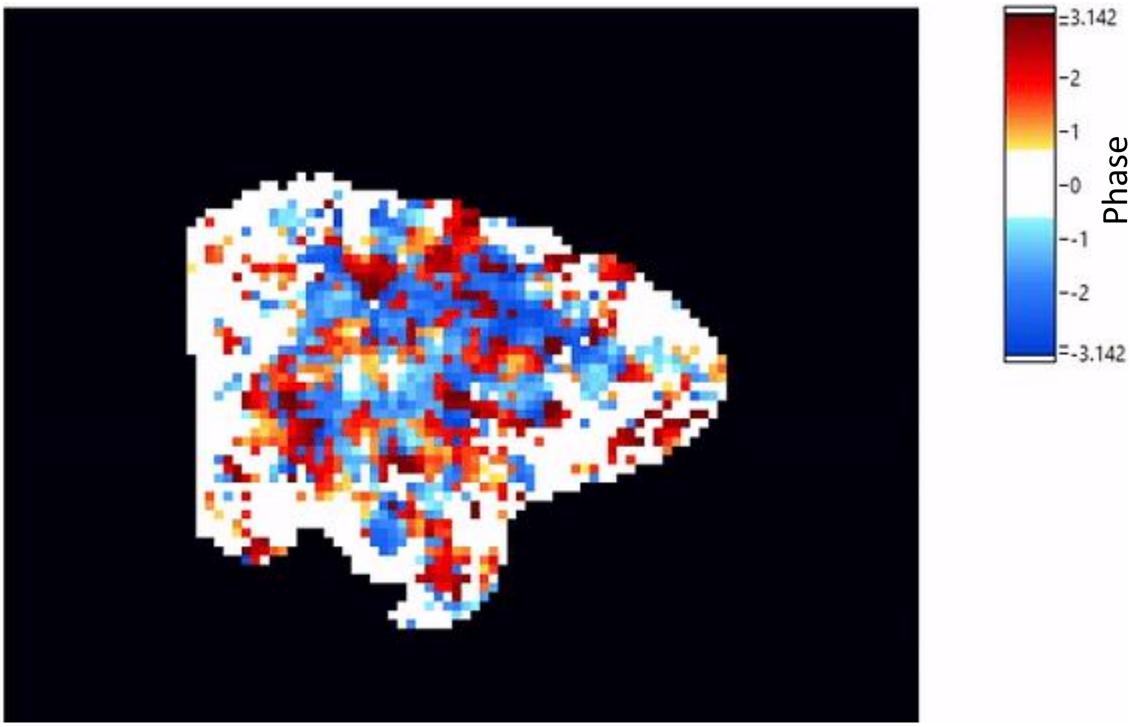

Supplementary Video. Phase video of cytosolic calcium level in a trabecula carneae from a human right atrial appendage. A video of phase changing over time, calculated using Hilbert-tranform. To improve visibility, the replay speed is slowed down 2-fold. To access video, please open Supplementary video file.

References

1. Borysova L, Ng YYH, Wragg ES, Wallis LE, Fay E, Ascione R, Dora KA. High spatial and temporal resolution Ca^{2+} imaging of myocardial strips from human, pig and rat. *Nat Protoc 2021 1610* 2021;**16**:4650–4675. DOI:10.1038/s41596-021-00590-6

2. Heijman J, Muna AP, Veleva T, Molina CE, Sutanto H, Tekook M, Wang Q, Abu-Taha IH, Gorka M, Künzel S, El-Armouche A, Reichenspurner H, Kamler M, Nikolaev V, Ravens U, Li N, Nattel S, Wehrens XHT, Dobrev D. Atrial Myocyte NLRP3/CaMKII Nexus Forms a Substrate for Postoperative Atrial Fibrillation. *Circ Res* 2020;**127**:1036–1055. DOI:10.1161/CIRCRESAHA.120.316710

3. Billings FT, Balaguer JM, Yu C, Wright P, Petracek MR, Byrne JG, Brown NJ, Pretorius M. Comparative Effects of Angiotensin Receptor Blockade and ACE Inhibition on the Fibrinolytic and Inflammatory Responses to Cardiopulmonary Bypass. *Clin Pharmacol Ther* 2012;**91**:1065–1073. DOI:10.1038/CLPT.2011.356
