# Supplementary figures and images for: Cytosolic calcium handling signature: integration with clinical predictors enhances prediction of post-operative atrial fibrillation
Source: Eur Heart J. 2025 Sep 9;47(13):1609–21. doi: 10.1093/eurheartj/ehaf609 (PMC13043196; doi:10.1093/eurheartj/ehaf609)

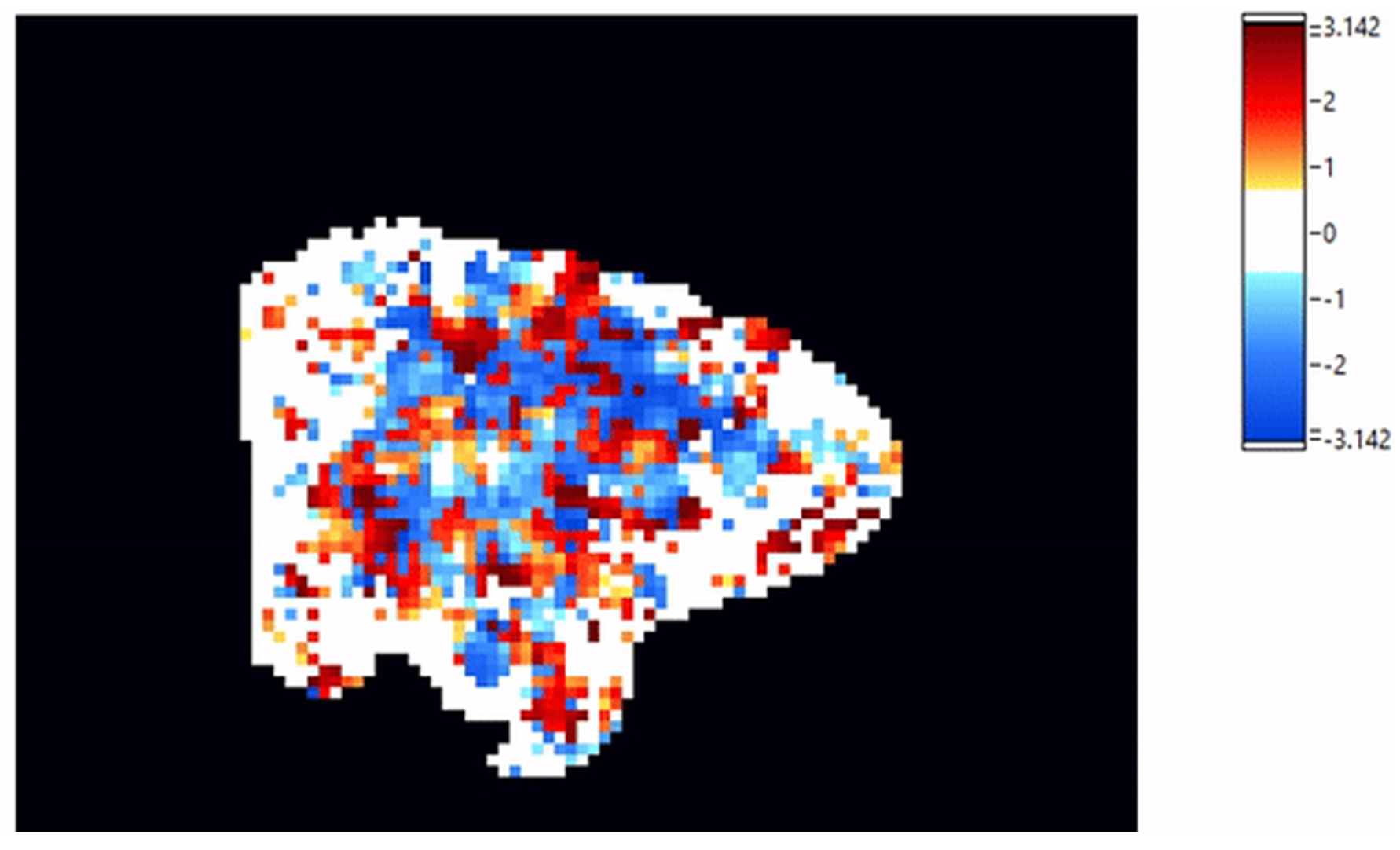

Supplement: ehaf609_Supplementary_Data [file ehaf609_supplementary_data.zip › Video (Still image).tif]
